# A Physiologically Based Pharmacokinetic Model for In Vivo Alpha Particle Generators Targeting Neuroendocrine Tumors in Mice

**DOI:** 10.3390/pharmaceutics13122132

**Published:** 2021-12-10

**Authors:** Nouran R. R. Zaid, Peter Kletting, Gordon Winter, Vikas Prasad, Ambros J. Beer, Gerhard Glatting

**Affiliations:** 1 Medical Radiation Physics, Department of Nuclear Medicine, Ulm University, 89081 Ulm, Germany; peter.kletting@uniklinik-ulm.de (P.K.); gerhard.glatting@uniklinik-ulm.de (G.G.); 2 Biophysics and Medical Imaging Program, Department of Biomedical Sciences, Faculty of Medicine and Health Sciences, An-Najah National University, Nablus 44839, Palestine; 3 Department of Nuclear Medicine, Ulm University, 89081 Ulm, Germany; gordon.winter@uni-ulm.de (G.W.); vikas.prasad@uniklinik-ulm.de (V.P.); ambros.beer@uniklinik-ulm.de (A.J.B.)

**Keywords:** murine PBPK model, neuroendocrine tumors, α-PRRT, in vivo alpha particle generators, [^212^Pb]Pb-DOTAMTATE

## Abstract

In vivo alpha particle generators have great potential for the treatment of neuroendocrine tumors in alpha-emitter-based peptide receptor radionuclide therapy (α-PRRT). Quantitative pharmacokinetic analyses of the in vivo alpha particle generator and its radioactive decay products are required to address concerns about the efficacy and safety of α-PRRT. A murine whole-body physiologically based pharmacokinetic (PBPK) model was developed for ^212^Pb-labeled somatostatin analogs (^212^Pb-SSTA). The model describes pharmacokinetics of ^212^Pb-SSTA and its decay products, including specific and non-specific glomerular and tubular uptake. Absorbed dose coefficients (ADC) were calculated for bound and unbound radiolabeled SSTA and its decay products. Kidneys received the highest ADC (134 Gy/MBq) among non-target tissues. The alpha-emitting ^212^Po contributes more than 50% to absorbed doses in most tissues. Using this model, it is demonstrated that α-PRRT based on ^212^Pb-SSTA results in lower absorbed doses in non-target tissue than α-PRRT based on ^212^Bi-SSTA for a given kidneys absorbed dose. In both approaches, the energies released in the glomeruli and proximal tubules account for 54% and 46%, respectively, of the total energy absorbed in kidneys. The ^212^Pb-SSTA-PBPK model accelerates the translation from bench to bedside by enabling better experimental design and by improving the understanding of the underlying mechanisms.

## 1. Introduction

Alpha-emitter-based peptide receptor radionuclide therapy (α-PRRT) using alpha-emitter-labeled somatostatin analogs (α-SSTA) is an efficacious treatment for metastatic inoperable neuroendocrine tumors (NET) [1]. Due to the high linear energy transfer (80–100 keV/µm), alpha particles have the potential to eradicate somatostatin receptor subtype 2 (SSTR2) expressing tumor cells by producing irreparable DNA double-strand breaks while sparing normal tissue [2]. As a consequence, α-PRRT has demonstrated its potential to reduce nephrotoxicity and bypass the radioresistance of NET to beta emitters in beta-emitter-based PRRT [3].

Several SSTA coupled to short-(^213^Bi) or long-lived alpha emitters (^225^Ac and ^212^Pb) are explored in preclinical and clinical settings to evaluate the efficacy and safety of α-SSTA for NET [1]. Studying the pharmacokinetics (PK) of in vivo alpha particle generators is challenging, especially with regard to the fate of the released radioactive products that are not easily accessible to direct measurements. A number of pharmacokinetic studies were based on blood samples [4], urine excretion, portable detectors and planar images to measure the effect of redistributed free radioactive decay products in the body [5,6,7]. As the injected therapeutic activity is much lower than the one used in diagnosis, quantitative imaging of short-lived alpha emitting daughters is limited by low spatial resolution and poor signal-to-noise ratio [8]. The use of theranostic pairs consisting of two chemically similar radionuclides (La^+3^ ions for Ac^+3^) or chemically identical radioisotopes (^203^Pb for ^212^Pb) represents a great advance, albeit more data are required to validate that concept [9,10]. For example, the biodistribution of the theranostic pairs may vary due to their different ionic radii [11,12], injected amounts [13] and half-lives [14].

Mathematical modeling may be the method of choice to describe the PK of each of the decay products of the in vivo alpha particle generators separately and simultaneously [15,16]. For this purpose, we developed a whole-body physiologically based pharmacokinetic (PBPK) model for mice to describe the PK of the in vivo alpha particle generator ^212^Pb-SSTA and its radioactive decay products in α-PRRT. The ^212^Pb-SSTA-PBPK model was evaluated using published biokinetic data of [^212^Pb]Pb-DOTAMTATE in mice bearing AR42J xenografts [4]. The absorbed doses due to bound and unbound radiolabeled SSTA in target and non-target tissues were calculated for different injected amounts and activities. In addition, the optimal tumor-to-kidneys ratio of long-lived conjugated ^212^Pb were calculated and compared to that of short-lived conjugated ^212^Bi.

## 2. Materials and Methods

### 2.1. ^212^Pb-SSTA-PBPK Model Structure

**Figure 1:**
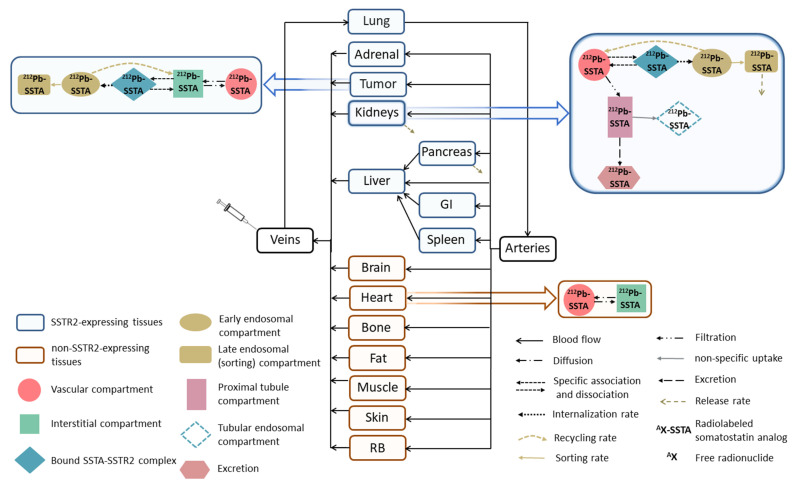
Scheme of the ^212^Pb-SSTA-PBPK model for mice. The model describes the PK of [^212^Pb]Pb-SSTA and the fate of its decay products in tissues. [^212^Pb]Pb-SSTA are distributed in the circulation to the vascular compartments of SSTR2-expressing and non-SSTR2-expressing tissues. After the diffusion of [^212^Pb]Pb-SSTA to the interstitial compartments, [^212^Pb]Pb-SSTA undergo specific binding followed by internalization to early endosomes where [^212^Pb]Pb-SSTA are either recycled back to the interstitial compartments or directed to the sorting compartments for degradation. Non-SSTR2-expressing tissues include only vascular and interstitial compartments. Each compartment in the ^212^Pb-SSTA-PBPK model represents the in vivo alpha particle generator model.

A whole-body PBPK model of ^212^Pb-SSTA in mice was developed and implemented using software tools SAAM II version 2.3 [17] (The Epsilon Group, TEG, Charlottesville, VA, USA) and Simbiology/MATLAB (MATLAB R2020a, The MathWorks, Inc., Natick, MA, USA). The structure of the ^212^Pb-SSTA-PBPK model describing the distribution of ^212^Pb-SSTA in mouse tissues via blood flow is illustrated in Figure 1. Relevant differential equations of the developed mathematical model maintain mass and blood flow conservation and are provided in supplement A. The transfer rates are described and their values are given in detail in Supplementary Material Table S1.

The compartmental model comprises (1) SSTR2-expressing tissues such as tumor, pancreas, kidneys, spleen, liver, adrenal gland (Ad), lung and gastrointestinal (GI) tract [18,19,20,21], (2) non-SSTR2-expressing tissues, namely muscle, heart, skin, brain, fat and remainder of body (RB).

**Figure 2:**
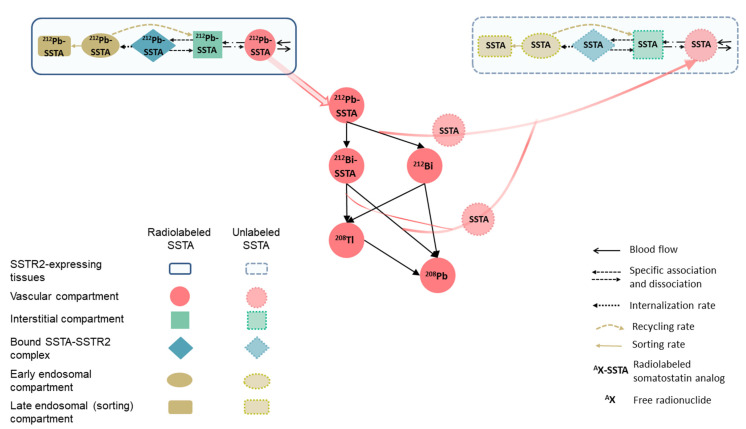
Scheme of the distribution of unlabeled SSTA in SSTR2-expressing tissue in the ^212^Pb-SSTA-PBPK model. The injected SSTA with radiolabeled SSTA, the SSTA produced after the dissociation of a fraction of ^212^Bi-chelator complexes after beta decay of ^212^Pb and the SSTA produced after alpha decay of ^212^Bi and ^212^Po are distributed in tissues via blood flow. The distributed SSTA were assumed to follow the same PK of radiolabeled SSTA and both compete on free binding sites. The structure of non-SSTR2-expressing tissues for unlabeled SSTA is the same as the one for radiolabeled SSTA.

The parameters of the ^212^Pb-SSTA-PBPK model are classified into mice-specific and ^212^Pb-SSTA-specific parameters with values taken from literature (Supplementary Material Table S1) [4,16,22,23,24,25,26,27,28,29,30,31,32,33,34,35,36,37]. Briefly, the main mice-specific parameters considered in the model are blood flow, tissue volumes, relevant volumes of tissue compartments (vascular, interstitial, early endosomes and sorting (late endosomes) compartments) and SSTR2 expression on cell membranes. Relevant biological mechanisms of ^212^Pb-SSTA (diffusion, SSTR2-specific and non-specific binding, internalization, recycling, sorting and excretion) are included in the model. In addition, the model takes into account the physicochemical properties of ^212^Pb-SSTA, such as physical decay rates and the chemical stability of the ^212^Bi-chelator complexes after beta decay of conjugated ^212^Pb [23]. The distribution of ^212^Pb-SSTA and unlabeled SSTA is described using two similar systems sharing the same physiological parameter values [16]. The two systems are linked by the competition of ^212^Pb-SSTA and unlabeled SSTA for free binding sites and the physical decay rates as shown in Figure 2.

**Figure 3:**
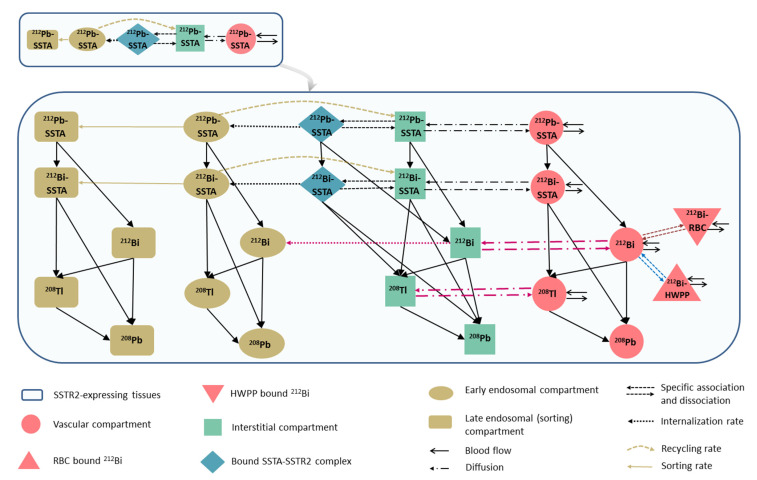
Detailed scheme of the structure of SSTR2-expressing tissue incorporating the in vivo alpha particle generator model and relevant pharmacokinetic parameters of free ^212^Bi. [^212^Pb]Pb-SSTA and [^212^Bi]Bi-SSTA are assumed to follow the same PK throughout the tissue. Free ^212^Bi in the vasculature binds linearly with RBC and HWPP followed by free ^212^Bi diffusion and uptake by tissue cells. The free daughter ^208^Tl is assumed to have the same permeability surface area product as that for free ^212^Bi so that it diffuses between interstitial and vascular compartments and redistributes in the circulation. The alpha-emitting ^212^Po decays at the site of its production.

Each compartment in the ^212^Pb-SSTA-PBPK model represents the in vivo alpha particle generator model described before [23]. The biodistribution of free ^212^Bi is described by integrating a PBPK model for free ^212^Bi into the ^212^Pb-SSTA-PBPK model [29]. In short, the ^212^Bi-PBPK model describes the interaction of free ^212^Bi with red blood cells (RBC), high molecular weight plasma proteins (HWPP) and intracellular biological thiols. The detailed compartmental structure of SSTR2-expressing tissues is presented in Figure 3.

The bound ^212^Pb-SSTA-SSTR2 complexes on cells surfaces are internalized to early endosomes (Figure 1) with a rate of 0.17 min^−1^ [35]. After internalization, a fraction of ^212^Pb-SSTA-SSTR2 complexes in early endosomes are dissociated followed by the recycling of SSTR2 to cell surfaces and the release of intact ^212^Pb-SSTA to the interstitial compartment with a transfer rate 0.05 min^−1^ [36]. The remaining ^212^Pb-SSTA-SSTR2 complexes in early endosomes are directed to the sorting compartment for degradation. The release rates of residualizing radiolabels or radiolabeled catabolites from cells were estimated (as described below).

A kidney model was developed to adequately describe the kinetics of ^212^Pb-SSTA in kidneys as the main excretion organ (upper right corner of Figure 1) [28]. As SSTR2 expression sites in mice kidneys were found in epithelial cells (podocytes) of the glomeruli and collecting ducts [18,38,39,40], the specific uptake of ^212^Pb-SSTA is modeled by connecting the compartment of bound ^212^Pb-SSTA-SSTR2 complexes directly to the vascular compartment of kidneys. The localization of SSTR2 in the collecting ducts was not considered in the kidney model because the reabsorption of the radiolabeled peptides beyond the convoluted proximal tubules is either small or absent [41]. Post-glomerular peritubular elimination was also not considered because proteins are not appreciably extracted through this elimination route as compared to the filtration pathway. Additionally, the post-glomerular elimination route is specific for small proteins, which have hormone-specific receptors located in the basolateral tubular cells [28,41]. As the non-specific uptake of radiolabeled peptides in the proximal tubule is mainly due to charge-related binding to megalin and/or cubilin receptors [42], fluid- and receptor-based endocytosis were integrated in the kidney model as a single compartment connected to the proximal tubules with a transfer rate 0.03 min^−1^ [43]. Although Behr et al. reported that some of the degradation products in renal tubules are transferred back into the bloodstream [44,45], this is not included in the model because of the limited number of data points and the relative short half-lives of ^212^Pb and all its daughters.

### 2.2. Model Evaluation, Parameters Estimation and Sensitivity Analyses

The performance of the developed ^212^Pb-SSTA-PBPK model was compared in SAAM II and Simbiology by running different simulations using the same parameterization and model inputs. The values of the ^212^Pb-SSTA-PBPK model parameters were estimated in both software tools by fitting the time–activity curves to [^212^Pb]Pb-DOTAMTATE biokinetics in AR42J-tumor bearing mice after injecting 0.0013 nmol (185 kBq) of [^212^Pb]Pb-DOTAMTATE to match the experimental condition (Supplementary Material Table S2) [4]. The computational settings in SAAM II were the Rosenbrock least-squares algorithm and a convergence criterion of 10^−4^. Model-based relative weighting was assigned for all data. The goodness-of-fit criteria were evaluated based on standard criteria reported, such as visual inspection of the fitted curves, coefficient of variation (CV < 50%) and off-diagonal values of the correlation matrix (−0.8 < CM < 0.8 for most elements) [46]. In Simbiology’s fitting settings, proportional error was used with the fminsearch function. The FunctionTolerance was 10^−14^, that is, the lower limit for the change in the value of the objective function during the fitting. Sensitivity analyses and a first evaluation of the predictive performance of the model were performed as described in detail in supplement D and supplement E, respectively.

### 2.3. Dosimetry and Simulations

The absorbed doses were calculated using the developed ^212^Pb-SSTA-PBPK model in Simbiology, because the number of compartments required for the absorbed dose calculations exceeds what could be practically provided by SAAM II. Absorbed dose coefficients (ADC) were calculated for the mouse tissues based on the MIRD formalism as follows [47]:

(1) $$ADC_{i}\left(T_{D}\right)=\frac{\sum_{x}D_{i}\left(T_{D}\right)}{A_{0}}={\tilde{a}}_{i}\left(T_{D}\right)\cdot \frac{\sum_{x}{∆}_{i}^{x}\cdot \varphi_{i}\left(E_{i}^{x}\right)}{M_{i}},$$

where $D_{i}\left(T_{D}\right)$ is the absorbed dose to tissue i of mass $M_{i}$ from emission type x; ${\tilde{a}}_{i}\left(T_{D}\right)$ is the total number of nuclear transitions in tissue i over the integration period $T_{D}\;$(10 · t_1/2_(^212^Pb) = 6384 min) divided by the administered activity $A_{0}$ (185 kBq of [^212^Pb]Pb-DOTAMTATE to match the experimental condition [4]),$\;{∆}_{i}^{x}$ is the mean energy absorbed in tissue i per nuclear transition of type x, $\varphi_{i}\left(E_{i}^{x}\right)$ is the fraction of emitted energy per nuclear transition absorbed in tissue i. $\varphi_{i}\left(E_{i}^{x}\right)$ is assumed to be equal to 1 as the target tissue is considered as the source tissue so that the emitted radiation is locally deposited [47]. Subsequently, the contributions of each radioactive decay product of [^212^Pb]Pb-DOTAMTATE to the total ADC were also determined for each tissue.

In addition, the absorbed doses were simulated for injected amounts of 0.017 × 10^−3^–0.079 nmol in steps of 10^1/6^ of [^212^Pb]Pb-DOTAMTATE, with injected activities chosen to lead to 23 Gy in kidneys. By injecting [^212^Bi]Bi-DOTAMTATE into the corresponding venous compartment in the ^212^Pb-SSTA-PBPK model with the same amounts, the doses absorbed by the tissues were calculated based on the non-generator equivalent [^212^Bi]Bi-DOTAMTATE. Additionally, total energies released in the glomeruli (the sum of energies released in renal vascular, bound SSTA-SSTR2 complexes, early endosomes, and sorting compartments) and proximal tubules (the sum of energies released in renal proximal tubules and tubular endosomal compartments) to deposit 23 Gy in kidneys were calculated following the administration of [^212^Pb]Pb-DOTAMTATE and [^212^Bi]Bi-DOTAMTATE.

## 3. Results

**Figure 4:**
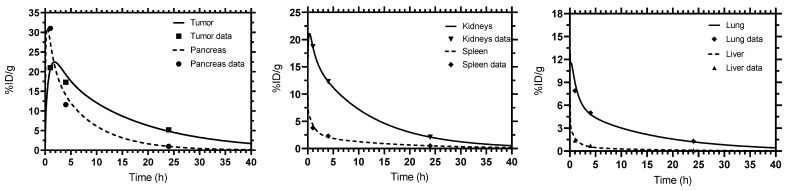
Fitted time–activity curves. Simbiology and SAAM II gave identical results.

The fitted time-activity curves generated by SAAM II and Simbiology for tumor, kidneys, pancreas, lung, spleen and liver show good fits. The results of the two software tools are in agreement and are equivalent for each tissue as shown in Figure 4. The values of the pharmacokinetic parameters estimated by fitting to the experimental data are given in Table 1. The CVs of the fitted parameters were less than 50% for all tissues. All elements of the correlation matrix of the fitted parameters were less than 0.8 except for tumor (perfusion rate and receptor density) and kidneys (release rate). Kidneys had the highest ADC among other non-target tissues (134 Gy/MBq) followed by pancreas (116 Gy/MBq) (Table 2). The contribution of ^212^Po to the total ADC was the highest in all tissues (more than 50% except in fat).

**Figure 5:**
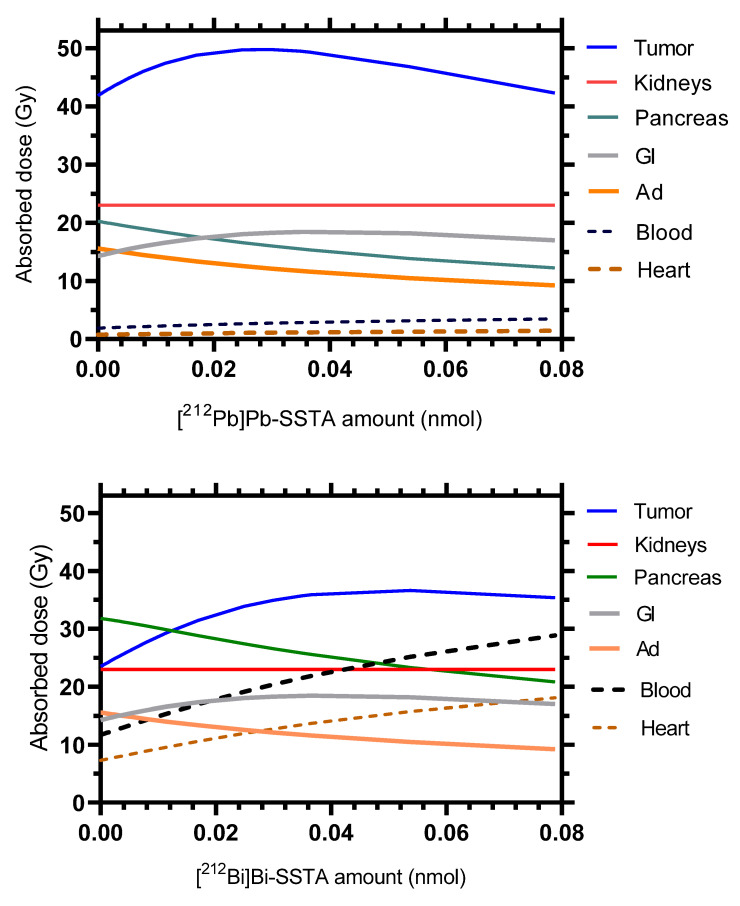
Therapeutic potencies of the in vivo alpha generator [^212^Pb]Pb-SSTA (upper panel) and [^212^Bi]Bi-SSTA (lower panel). The absorbed doses in different mouse tissues were calculated for different injected amounts, with activity chosen such that kidneys always received a dose of 23 Gy. The absorbed doses in the other tissues of the model are presented in Supplementary Material Figure S1.

**Figure 6:**
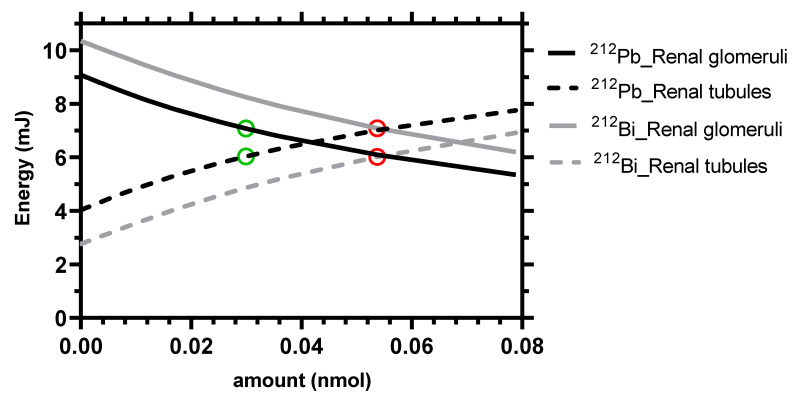
Energy absorbed in the renal glomeruli and proximal tubules resulting in 23 Gy in the kidneys depending on the administered amounts of [^212^Pb]Pb-SSTA or [^212^Bi]Bi-SSTA. The green and red circles indicate the energies released at the optimal amounts of [^212^Pb]Pb-SSTA and [^212^Bi]Bi-SSTA, respectively.

For a given value of 23 Gy in kidneys, the maximum absorbed dose in the tumor (49.8 Gy) was reached with the administration of 0.03 nmol of [^212^Pb]Pb-DOTAMTATE as shown in Figure 5. For the non-generator equivalent [^212^Bi]Bi-DOTAMTATE, only 36.6 Gy can be reached in the tumor at the optimal amount of 0.05 nmol of [^212^Bi]Bi-DOTAMTATE. Figure 6 shows that the energies released in the glomeruli and proximal tubules account for 54% and 46%, respectively, of the total energy absorbed in kidneys at the simulated optimal amounts of both [^212^Pb]Pb-DOTAMTATE and [^212^Bi]Bi-DOTAMTATE.

## 4. Discussion

In this study, a ^212^Pb-SSTA-PBPK model was developed to investigate the PK and the dosimetry of ^212^Pb-SSTA and its cytotoxic radioactive products targeting SSTR2-expressing tissues. The main aim of this work was to describe a first developed complex whole-body PBPK model for ^212^Pb-based in vivo alpha particle generators and its radioactive decay products. The complexity of the model arises from combining different mathematical models, that is, a ^212^Pb-PBPK model for conjugated ^212^Pb, a PBPK model for free ^212^Bi, and the in vivo alpha particle generator model. The acceptable initial evaluations of the model (sensitivity analysis and predictive performance) in supplements D and E place the model in context of generating hypotheses that support next/future studies. The developed ^212^Pb-SSTA-PBPK model underpins the higher potential of the in vivo alpha particle generator [^212^Pb]Pb-DOTAMTATE in α-PRRT in comparison to its non-generator equivalent [^212^Bi]Bi-DOTAMTATE as demonstrated in Figure 5 and Figure 6. For a given value of 23 Gy in kidneys, [^212^Pb]Pb-DOTAMTATE delivers a higher dose using a lower optimal amount (49.8 Gy within 0.03 nmol) than [^212^Bi]Bi-DOTAMTATE (36.6 Gy within 0.05 nmol). In addition, for [^212^Pb]Pb-DOTAMTATE, all absorbed doses in non-target tissues are less than kidneys absorbed dose.

The distinction between absorbed doses to glomeruli (the radiation-sensitive functional unit for late damage [48]) and proximal tubules is important for renal toxicity [49,50]. Figure 6 shows that the specific uptake in the kidneys results in more energy being released in the glomeruli than in the proximal tubules, except for larger amounts when SSTR2 is saturated. Due to the shorter half-life, [^212^Bi]Bi-DOTAMTATE injection results in higher energies released in the glomeruli than by [^212^Pb]Pb-DOTAMTATE injection.

For mice, pancreas can be the second organ at risk after kidneys in α-PRRT because of its high ADC (116 Gy/MBq, Table 2), which is in line with the findings of Norenberg et al. and Kimura et al. [38,51]. Additionally, Table 2 demonstrates the ability of the ^212^Pb-SSTA-PBPK model to address the concerns regarding the fate of the redistributed radioactive product of [^212^Pb]Pb-DOTAMTATE (conjugated and free radionuclides). The largest contribution to the total tissue absorbed dose is related to conjugated alpha emitters, mainly conjugated ^212^Po. The absorbed doses in non-SSTR2 expressing tissues are due to the distribution of [^212^Pb]Pb-DOTAMTATE and its progeny mainly in the vascular and interstitial compartments. Of note, the reported high uptake rate of free ^212^Bi in bone explains the high contribution of free radionuclides to total absorbed dose in Table 2 [29].

Sites and levels of SSTR2 expression differ between species [38]. Intriguingly, the estimated SSTR2 densities in kidneys (3.52 ± 0.07 nmol/L) and tumor (11.7 ± 3.0 nmol/L) in AR42J-tumor bearing mice were in the reported ranges of renal (2.3–7.1 nmol/L) and tumor (7–16 nmol/L) SSTR2 densities in humans [16]. The relatively high SSTR2 density in pancreas, which is not observed in humans, can be related to the additional expression of SSTR2A in murine pancreatic acinar cells [38]. In addition, the difference in the microanatomical structure of spleen in mice and humans may contribute to the difference in the estimated SSTR2 densities in mice (0.73 ± 0.07 nmol/L) and the reported range in humans (3.9–8.7 nmol/L) [16]. In contrast to mice, the red pulp in human spleen has sheathed capillaries, composed of macrophages and B lymphocytes which are reported to express SSTR2 mRNA [52,53]. These differences in the levels of SSTR2 expression should be considered in the translation from mice to human PBPK models.

Incorporating internalization, recycling, and sorting mechanisms is important to adequately reflect the SSTR2 densities and to describe the concentrations of radiolabeled peptides both inside and outside of cells [36]. It is important to highlight that the fraction of intact [^212^Pb]Pb-SSTA-SSTR2 complexes in early endosomes is sorted for degradation with a rate that solely depends on the type of receptors and targeting vectors. Preliminary fits revealed that the release rates of the degradation products of [^212^Pb]Pb-DOTAMTATE were zero except for kidneys and pancreas. Therefore, [^212^Pb]Pb-DOTAMTATE as a radiometal-chelated SSTA was assumed to be retained inside the cells after being routed to lysosomes in the investigated time interval. Vegt et al. and others showed that some residualizing radiolabels or radiolabeled catabolites cannot cross the lysosomal membrane, thus stay trapped in lysosomes delivering high radiation doses in the renal tubules and glomeruli [36,54,55,56]. On the other hand, because of the relatively short half-life of ^212^Pb (t_1/2_ = 10 h) in comparison to beta emitters used in PRRT, such as ^177^Lu (t_1/2_ = 6.6 d), the experimental data do not provide enough information to fit the release rate. However, as the time scale of the release rate is very small compared to the half-life of ^212^Pb, setting the release rate to zero does not affect the results of the simulations. The fitted release rate of pancreas was high and that may be because secretory acinar pancreatic cells have distinct functions that may require a specific and polarized SSTR2 distribution and trafficking as reported by Waser et al. [57].

The developed ^212^Pb-SSTA-PBPK model for mice can be employed for broad future investigations. Simulating concentration–time profiles can help in the selection of optimal sampling schedules and optimize dose regimens in different study populations, including humans [16]. The separation between radiopharmaceutical-specific and physiological parameters in the ^212^Pb-SSTA-PBPK model allows optimal activities to be administered for individualized dosimetry, thereby minimizing risks [15]. Additionally, the ^212^Pb-SSTA-PBPK model enables to calculate the absorbed doses in tissue compartments, that is, sub-organ doses. Therefore, using virtual preclinical studies based on mathematical modeling can be a first step for optimizing treatment planning in α-PRRT.

In addition, optimal values for global pharmacokinetic parameters, such as ligand affinities, internalization and sorting rates, can be simulated using the ^212^Pb-SSTA-PBPK model for newly developed in vivo alpha particle generators in α-PRRT. The model can be used to study the effect of using different chelators with different dissociation fractions of ^212^Bi-chelator complexes after beta decay of conjugated ^212^Pb [23].

Notably, the kidney model provides a full description of the relevant mechanisms underlying SSTA uptake in kidneys. The specific and non-specific renal uptake implemented in the model may explain the partial blocking of renal uptake achieved by the co-administration of amino acids lysine and arginine or unlabeled SSTA [37]. The kidney model structure can be integrated in a human PBPK model and contribute to future work aiming to reduce the probability of long-term renal toxicity in PRRT for NET.

Although strong background information was integrated during model development, and the initial evaluation of the model was acceptable, clearly, more well-characterized data will be required in the future for further improvements and testing of the predictive performance in order to achieve regulatory impact.

## 5. Conclusions

The developed ^212^Pb-SSTA-PBPK model quantitatively determines the non-measurable PK of ^212^Pb-SSTA and its radioactive decay products in mice and describes the specific and non-specific renal uptakes by the kidney model. The model allows absorbed doses to be calculated from unbound, bound, and internalized ^212^Pb-SSTA and the radioactive decay products in the whole body. Additionally, optimal injected amounts and activities can be determined using simulations to better design preclinical experiments in α-PRRT. Furthermore, the ^212^Pb-SSTA-PBPK model shows that ^212^Pb-SSTA-based α-PRRT results in a higher tumor-to-kidneys ratio than ^212^Bi-SSTA-based α-PRRT for a given kidneys absorbed dose.

## Figures and Tables

**Table 1:** Pharmacokinetic parameter values obtained from the ^212^Pb-SSTA-PBPK model fit to experimental data [4].

Parameter (Unit)	Tissue	SAAM II	Simbiology
		Estimated Value	
SSTR2 density (nmol·L^−1^)	Kidneys	3.52 ± 0.07	3.52
	Liver	0.17 ± 0.04	0.16
	Spleen	0.73 ± 0.07	0.73
	Lung	1.77 ± 0.14	1.77
	Pancreas	6.18 ± 0.94	6.18
	Tumor	11.73 ± 2.97	11.73
Perfusion (mL·min^−1^·g^−1^)	Tumor	0.09 ± 0.03	0.09
Sorting rate (min^−1^)	SSTR2-expressing tissues	0.0076 ± 0.0003	0.0076
Release rate (min^−1^)	Pancreas	0.0011 ± 0.0002	0.0011
	Kidneys	0.000380 ± 0.000001	0.000380

**Table 2:** Total absorbed dose coefficients (ADC) and the contributions of the in vivo alpha particle generator [^212^Pb]Pb-DOTAMTATE and its radioactive decay products to the total absorbed doses in SSTR2-expressing and non-SSTR2-expressing tissues.

Tissue		ADC (Gy/MBq)	Contribution to the Total Absorbed Dose per Tissue (%)							
			Conjugated Radionuclides				Free Radionuclides			
			^212^Pb	^212^Bi	^212^Bi	^212^Po	^212^Bi	^208^Tl	^212^Bi	^212^Po
			Beta		Alpha		Beta		Alpha	
SSTR2-expressing tissues	Tumor	248	5	11	18	45	2	6	3	8
	Kidneys	134	6	11	18	45	2	6	3	8
	Pancreas	116	6	11	17	44	2	6	4	9
	Liver	9	5	9	14	35	5	6	7	19
	Lung	65	6	11	18	45	2	6	3	8
	Spleen	28	6	11	17	44	2	6	4	10
	Ad	89	6	11	18	45	2	6	3	9
	GI	85	5	11	18	45	2	6	3	9
Non-SSTR2-expressing tissues	Skin	1.8	9	11	16	42	2	6	4	9
	Bone	2.7	3	3	5	14	10	7	16	41
	Muscle	0.6	11	11	18	46	1	7	2	4
	Heart	3.6	2	14	23	58	0	1	0	1
	Brain	0.1	12	11	17	43	2	6	3	7
	Fat	1.0	9	9	14	36	1	28	1	3
	RB	1.0	11	11	18	45	1	6	2	5

## Data Availability

The used data, additional to those in the supplement are available from the corresponding author on reasonable request.

## Supplementary Materials

The following are available online at https://www.mdpi.com/article/10.3390/pharmaceutics13122132/s1, Figure S1: Comparison of the therapeutic potency of the in vivo alpha generator [^212^Pb]Pb-SSTA (upper panel) and [^212^Bi]Bi-SSTA (lower panel), Figure S2: Kidneys sensitivity plots, Figure S3: Tumor sensitivity plots, Figure S4: Pancreas sensitivity plots, Table S1: [^212^Pb]Pb-SSTA-PBPK model parameters, Table S2: Non-decay corrected %ID/g for [^212^Pb]Pb-DOTAMTATE in different tissues of unanesthetized AR42J-bearing mice at different time points, Table S3: The predicted values of %ID/g for relevant tissues after injecting 5 µCi of ^212^Pb-DOTAMTATE with a specific activity of 2.4 µCi/ng.
