# Supplementary material for: A Physiologically Based Pharmacokinetic Model for In Vivo Alpha Particle Generators Targeting Neuroendocrine Tumors in Mice
Source: Pharmaceutics. 2021 Dec 10;13(12):2132. doi: 10.3390/pharmaceutics13122132 (PMC8703774; doi:10.3390/pharmaceutics13122132)
Supplement: Supplementary file 1 [file pharmaceutics-13-02132-s001.zip › pharmaceutics-1443721-supplementary.pdf]

pharmaceutics

Supplementary Material

A Physiologically Based Pharmacokinetic Model for In Vivo Alpha Particle Generators Targeting Neuroendocrine Tumors in Mice

Nouran R. R. Zaid [1,2,*], Peter Kletting [1,3], Gordon Winter [3], Vikas Prasad [3], Ambros J. Beer [3] and Gerhard Glatting [1,3]

[1] Medical Radiation Physics, Department of Nuclear Medicine, Ulm University, 89081 Ulm, Germany; peter.kletting@uniklinik-ulm.de (P.K.); gerhard.glatting@uniklinik-ulm.de (G.G.)
[2] Biophysics and Medical Imaging Program, Department of Biomedical Sciences, Faculty of Medicine and Health Sciences, An-Najah National University, Nablus, Palestine
[3] Department of Nuclear Medicine, Ulm University, 89081 Ulm, Germany; gordon.winter@uni-ulm.de (G.W.); vikas.prasad@uniklinik-ulm.de (V.P.); ambros.beer@uniklinik-ulm.de (A.J.B.)
* Correspondence: nouran.zaid@uni-ulm.de

Supplement A: Model equations and parameters
Supplement B: Biokinetic data for fitting
Supplement C: Therapeutic ratios of all tissues using ^{212}Pb-SSTA-PBPK model
Supplement D: Sensitivity analyses
Supplement E: Evaluation of the predictive performance of the ^{212}Pb-SSTA-PBPK model

Supplement A: Model equations and parameters

PBPK model equations

The following differential equations describe the pharmacokinetics of ^{212}Pb-labeled and ^{212}Bi-labeled somatostatin analogs, that is, ^{212}Pb-SSTA and ^{212}Bi-SSTA. Also, the pharmacokinetics of free nuclides, namely ^{212}Bi, ^{208}Tl and ^{208}Pb was described in SSTR2-expressing tissue i, which include tumor, pancreas, spleen, liver, adrenal and gastrointestinal tract, of ^{212}Pb-SSTA-PBPK model. The vascular and interstitial compartments of non-SSTR2-expressing tissues have similar differential equations as those of SSTR2-expressing tissues. All model parameters are defined in Table S1.

Differential equations for the *in vivo* alpha particle generator in the vascular compartment of SSTR2-expressing tissues except lung and kidneys:

$$\frac{d(^{212}Pb\text{-}SSTA)_{v,i}}{dt} = Fi \cdot \left(\frac{(^{212}Pb\text{-}SSTA)_{Art}}{V_{Art}} - \frac{(^{212}Pb\text{-}SSTA)_{v,i}}{V_{v,i}}\right) + PS_{i\,P} \cdot \left(\frac{(^{212}Pb\text{-}SSTA)_{inters,i}}{V_{inters,i}} - \frac{(^{212}Pb\text{-}SSTA)_{v,i}}{V_{v,i}}\right) - \lambda_{phy}(^{212}Pb) \cdot (^{212}Pb\text{-}SSTA)_{v,i} \quad (1)$$

$$\frac{d(^{212}Bi\text{-}SSTA)_{v,i}}{dt} = Fi \cdot \left(\frac{(^{212}Bi\text{-}SSTA)_{Art}}{V_{Art}} - \frac{(^{212}Bi\text{-}SSTA)_{v,i}}{V_{v,i}}\right) + PS_{,i\,P} \cdot \left(\frac{(^{212}Bi\text{-}SSTA)_{inters,i}}{V_{inters,i}} - \frac{(^{212}Bi\text{-}SSTA)_{v,i}}{V_{v,i}}\right) - \lambda_{phy}(^{212}Bi) \cdot (^{212}Bi\text{-}SSTA)_{v,i} + \lambda_{phy,gr}(^{212}Pb) \cdot (^{212}Pb\text{-}SSTA)_{v,i} \quad (2)$$

$$\frac{d(^{212}Bi)_{v,i}}{dt} = Fi \cdot \left(\frac{(^{212}Bi)_{Art}}{V_{Art}} - \frac{(^{212}Bi)_{v,i}}{V_{v,i}}\right) + PS_{,i}f \cdot \left(\frac{(^{212}Bi)_{inters,i}}{V_{inters,i}} - \frac{(^{212}Bi)_{v,i}}{V_{v,i}}\right) + (^{212}Bi\text{-}HWPP\text{-}diss) \cdot (^{212}Bi\text{-}HWPP)_{v,i} - (^{212}Bi\text{-}HWPP\text{-}ass) \cdot (^{212}Bi)_{v,i} + (^{212}Bi\text{-}RBC\text{-}diss) \cdot (^{212}Bi\text{-}RBC)_{v,i} - (^{212}Bi\text{-}RBC\text{-}ass) \cdot (^{212}Bi)_{v,i} - \lambda_{phy}(^{212}Bi) \cdot (^{212}Bi)_{v,i} + \lambda_{phy,ex}(^{212}Pb) \cdot (^{212}Pb\text{-}SSTA)_{v,i} \quad (3)$$

$$\frac{d(^{212}Bi\text{-}HWPP)_{v,i}}{dt} = F_i \cdot \left(\frac{(^{212}Bi\text{-}HWPP)_{Art}}{V_{Art}} - \frac{(^{212}Bi\text{-}HWPP)_{v,i}}{V_{v,i}}\right) + (^{212}Bi\text{-}HWPP\text{-}ass) \cdot (^{212}Bi)_{v,i} - (^{212}Bi\text{-}HWPP\text{-}diss) \cdot (^{212}Bi\text{-}HWPP)_{v,i} - \lambda_{phy}(^{212}Bi) \cdot (^{212}Bi\text{-}HWPP)_{v,i} \quad (4)$$

$$\frac{d(^{212}Bi\text{-}RBC)_{v,i}}{dt} = F_i \cdot \left(\frac{(^{212}Bi\text{-}RBC)_{Art}}{V_{Art}} - \frac{(^{212}Bi\text{-}RBC)_{v,i}}{V_{v,i}}\right) + (^{212}Bi\text{-}RBC\text{-}ass) \cdot (^{212}Bi)_{v,i} - (^{212}Bi\text{-}RBC\text{-}diss) \cdot (^{212}Bi\text{-}RBC)_{v,i} - \lambda_{phy}(^{212}Bi) \cdot (^{212}Bi\text{-}RBC)_{v,i} \quad (5)$$

$$\frac{d(^{208}Tl)_{v,i}}{dt} = F_i \cdot \left(\frac{(^{208}Tl)_{Art}}{V_{Art}} - \frac{(^{208}Tl)_{v,i}}{V_{v,i}}\right) + PS_{,i}f \cdot \left(\frac{(^{208}Tl)_{inters,i}}{V_{inters,i}} - \frac{(^{208}Tl)_{v,i}}{V_{v,i}}\right) + \lambda_{phy,\alpha}(^{212}Bi) \cdot [(^{212}Bi\text{-}SSTA)_{v,i} + (^{212}Bi)_{v,i} + (^{212}Bi\text{-}HWPP)_{v,i} + (^{212}Bi\text{-}RBC)_{v,i}] - \lambda_{phy}(^{208}Tl) \cdot (^{208}Tl)_{v,i} \quad (6)$$

$$\frac{d(^{208}Pb)_{v,i}}{dt} = \lambda_{phy,\beta}(^{212}Bi). [(^{212}Bi\text{-}SSTA)_{v,i} + (^{212}Bi)_{v,i} + (^{212}Bi\text{-}HWPP)_{v,i} + (^{212}Bi\text{-}RBC)_{v,i}] + \lambda_{phy}(^{208}Tl) \cdot (^{208}Tl)_{v,i} \quad (7)$$

Differential equations for the *in vivo* alpha particle generator in the vascular compartment of lung:

$$\frac{d(^{212}Pb\text{-}SSTA)_{v,Lun}}{dt} = F_{Lun} \cdot \left(\frac{(^{212}Pb\text{-}SSTA)_{Vein}}{V_{Vein}} - \frac{(^{212}Pb\text{-}SSTA)_{v,Lun}}{V_{v,Lun}}\right) + PS_{Lun,P} \cdot \left(\frac{(^{212}Pb\text{-}SSTA)_{inters,Lun}}{V_{inters,Lun}} - \frac{(^{212}Pb\text{-}SSTA)_{v,Lun}}{V_{v,Lun}}\right) - \lambda_{phy}(^{212}Pb) \cdot (^{212}Pb\text{-}SSTA)_{v,Lun} \quad (8)$$

$$\frac{d(^{212}Bi\text{-}SSTA)_{v,Lun}}{dt} = F_{Lun} \cdot \left(\frac{(^{212}Bi\text{-}SSTA)_{Vein}}{V_{Vein}} - \frac{(^{212}Bi\text{-}SSTA)_{v,Lun}}{V_{v,Lun}}\right) + PS_{Lun,P} \cdot \left(\frac{(^{212}Bi\text{-}SSTA)_{inters,Lun}}{V_{inters,Lun}} - \frac{(^{212}Bi\text{-}SSTA)_{v,Lun}}{V_{v,Lun}}\right) - \lambda_{phy}(^{212}Bi) \cdot (^{212}Bi\text{-}SSTA)_{v,Lun} + \lambda_{phy,gr}(^{212}Pb) \cdot (^{212}Pb\text{-}SSTA)_{v,Lun} \quad (9)$$

$$\frac{d(^{212}Bi)_{v,Lun}}{dt} = F_{Lun} \cdot \left(\frac{(^{212}Bi)_{Vein}}{V_{Vein}} - \frac{(^{212}Bi)_{v,Lun}}{V_{v,Lun}}\right) + PS_{Lun,f} \cdot \left(\frac{(^{212}Bi)_{inters,Lun}}{V_{inters,Lun}} - \frac{(^{212}Bi)_{v,Lun}}{V_{v,Lun}}\right)$$
$$+ (^{212}Bi\text{-}HWPP\text{-}diss) \cdot (^{212}Bi\text{-}HWPP)_{v,Lun} - (^{212}Bi\text{-}HWPP\text{-}ass) \cdot (^{212}Bi)_{v,Lun}$$
$$+ (^{212}Bi\text{-}RBC\text{-}diss) \cdot (^{212}Bi\text{-}RBC)_{v,Lun} - (^{212}Bi\text{-}RBC\text{-}ass) \cdot (^{212}Bi)_{v,Lun}$$
$$- \lambda_{phy}(^{212}Bi) \cdot (^{212}Bi)_{v,Lun} + \lambda_{phy,ex}(^{212}Pb) \cdot (^{212}Pb\text{-}SSTA)_{v,Lun} \quad \textbf{(10)}$$

$$\frac{d(^{212}Bi\text{-}HWPP)_{v,Lun}}{dt} = F_{Lun} \cdot \left(\frac{(^{212}Bi\text{-}HWPP)_{Vein}}{V_{Vein}} - \frac{(^{212}Bi\text{-}HWPP)_{v,Lun}}{V_{v,Lun}}\right) + (^{212}Bi\text{-}HWPP\text{-}ass) \cdot (^{212}Bi)_{v,Lun}$$
$$- (^{212}Bi\text{-}HWPP\text{-}diss) \cdot (^{212}Bi\text{-}HWPP)_{v,Lun} - \lambda_{phy}(^{212}Bi) \cdot (^{212}Bi\text{-}HWPP)_{v,Lun} \quad \textbf{(11)}$$

$$\frac{d(^{212}Bi\text{-}RBC)_{v,Lun}}{dt} = F_{Lun} \cdot \left(\frac{(^{212}Bi\text{-}RBC)_{Vein}}{V_{Vein}} - \frac{(^{212}Bi\text{-}RBC)_{v,Lun}}{V_{v,i}}\right) + (^{212}Bi\text{-}RBC\text{-}ass) \cdot (^{212}Bi)_{v,Lun}$$
$$-(^{212}Bi\text{-}RBC\text{-}diss) \cdot (^{212}Bi\text{-}RBC)_{v,Lun} - \lambda_{phy}(^{212}Bi) \cdot (^{212}Bi\text{-}RBC)_{v,Lun} \quad \textbf{(12)}$$

$$\frac{d(^{208}Tl)_{v,Lun}}{dt} = F_{Lun} \cdot \left(\frac{(^{208}Tl)_{Vein}}{V_{Vein}} - \frac{(^{208}Tl)_{v,Lun}}{V_{v,Lun}}\right) + PSLun_{,f} \cdot \left(\frac{(^{208}Tl)_{inters,Lun}}{V_{inters,Lun}} - \frac{(^{208}Tl)_{v,Lun}}{V_{v,Lun}}\right) +$$
$$\lambda_{phy,\alpha}(^{212}Bi) \cdot [(^{212}Bi\text{-}SSTA)_{v,Lun} + (^{212}Bi)_{v,Lun} + (^{212}Bi\text{-}HWPP)_{v,Lun} + (^{212}Bi\text{-}RBC)_{v,Lun}]$$
$$-\lambda_{phy}(^{208}Tl) \cdot (^{208}Tl)_{v\,Lun,} \quad \textbf{(13)}$$

$$\frac{d(^{208}Pb)_{v,Lun}}{dt} = \lambda_{phy,\beta}(^{212}Bi).\ [(^{212}Bi\text{-}SSTA)_{v,Lun} + (^{212}Bi)_{v,Lun} + (^{212}Bi\text{-}HWPP)_{v,Lun} + (^{212}Bi\text{-}RBC)_{v,Lun}]$$
$$+ \lambda_{phy}(^{208}Tl) \cdot (^{208}Tl)_{v,Lun} \quad \textbf{(14)}$$

Differential equations for the *in vivo* alpha particle generator in the interstitial compartment of SSTR2-expressing tissues:

$$\frac{d(^{212}Pb\text{-}SSTA)_{inters,i}}{dt} = PS_{,i}\,P \cdot \left(\frac{(^{212}Pb\text{-}SSTA)_{v,i}}{V_{v,i}} - \frac{(^{212}Pb\text{-}SSTA)_{inters,i}}{V_{inters,i}}\right) + \lambda_{rec} \cdot (^{212}Pb\text{-}SSTA)_{endo,i}$$
$$+koff \cdot (^{212}Pb\text{-}SSTA)_{bound,i} - \frac{k_{on}}{V_{inters,i}} \cdot Ri \cdot (^{212}Pb\text{-}SSTA)_{inters,i}$$
$$- \lambda_{phy}(^{212}Pb) \cdot (^{212}Pb\text{-}SSTA)_{inters,i} \quad \textbf{(15)}$$

$$\frac{d(^{212}Bi\text{-}SSTA)_{inters,i}}{dt} = PS_{,i}\,P \cdot \left(\frac{(^{212}Bi\text{-}SSTA)_{v,i}}{V_{v,i}} - \frac{(^{212}Bi\text{-}SSTA)_{inters,i}}{V_{inters,i}}\right) + \lambda_{\mathrm{rec}} \cdot (^{212}Bi\text{-}SSTA)_{endo,i}$$
$$+koff \cdot (^{212}Bi\text{-}SSTA)_{bound,i} - \frac{k_{on}}{V_{inters,i}} \cdot Ri \cdot (^{212}Bi\text{-}SSTA)_{inters,i}$$
$$-\lambda_{phy,gr}(^{212}Pb) \cdot (^{212}Pb\text{-}SSTA)_{inters,i} \tag{16}$$

$$\frac{d(^{212}Bi)_{inters,i}}{dt} = PS_{,i}\,f \cdot \left(\frac{(^{212}Bi)_{v,i}}{V_{v,i}} - \frac{(^{212}Bi)_{inters,i}}{V_{inters,i}}\right) - \lambda_{phy}(^{212}Bi) \cdot (^{212}Bi)_{inters,i}$$
$$+\lambda_{phy,ex}(^{212}Pb).[(^{212}Pb\text{-}SSTA)_{inters,i} + (^{212}Pb\text{-}SSTA)_{bound,i}] \tag{17}$$

$$\frac{d(^{208}Tl)_{inters,i}}{dt} = PS_{,i}\,f \cdot \left(\frac{(^{208}Tl)_{v,i}}{V_{v,i}} - \frac{(^{208}Tl)_{inters,i}}{V_{inters,i}}\right) + \lambda_{phy,\alpha}(^{212}Bi).\,[(^{212}Bi\text{-}SSTA)_{inters,i}$$
$$+ (^{212}Bi)_{inters,i} +$$
[illegible] (18)

$$\frac{d(^{208}Pb)_{inters,i}}{dt} = \lambda_{phy,\beta}(^{212}Bi) \cdot [(^{212}Bi\text{-}SSTA)_{inters,i} + (^{212}Bi)_{inters,i} + (^{212}Bi\text{-}SSTA)_{bound,i}]$$
$$+\lambda_{phy}(^{208}Tl).(^{208}Tl)_{inters,i} \tag{19}$$

Differential equations for the *in vivo* alpha particle generator for the radiolabeled SSTA-SSTR2 complexes on cell surfaces of SSTR2-expressing tissues:

$$\frac{d(^{212}Pb\text{-}SSTA)_{bound,i}}{dt} = -k_{off} \cdot (^{212}Pb\text{-}SSTA)_{bound,i} + \frac{k_{on}}{V_{inters,i}} \cdot Ri \cdot (^{212}Pb\text{-}SSTA)_{inters,i}$$
$$-\lambda_{intern} \cdot (^{212}Pb\text{-}SSTA)_{bound,i} - \lambda_{phy}(^{212}Pb) \cdot (^{212}Pb\text{-}SSA)_{bound,i} \tag{20}$$

$$\frac{d(^{212}Bi\text{-}SSTA)_{bound,i}}{dt} = -k_{off} \cdot (^{212}Bi\text{-}SSTA)_{bound,i} + \frac{k_{on}}{V_{inters,i}} \cdot Ri \cdot (^{212}Bi\text{-}SSTA)_{inters,i}$$
$$-\lambda_{intern} \cdot (^{212}Bi\text{-}SSTA)_{bound,i} + \lambda_{phy,gr}(^{212}Pb) \cdot (^{212}Pb\text{-}SSTA)_{bound,i}$$
$$-\lambda_{phy}(^{212}Bi) \cdot (^{212}Bi\text{-}SSTA)_{bound,i} \tag{21}$$

Differential equations for the *in vivo* alpha particle generator in the endosomal compartment of SSTR2-expressing tissues:

$$\frac{d(^{212}Pb\text{-}SSTA)_{endo,i}}{dt} = \lambda_{intern} \cdot (^{212}Pb\text{-}SSTA)_{bound,i} - \lambda_{rec} \cdot (^{212}Pb\text{-}SSTA)_{endo,i} - \lambda_{sort} \cdot (^{212}Pb\text{-}SSTA)_{endo,i} - \lambda_{phy}(^{212}Pb) \cdot (^{212}Pb\text{-}SSTA)_{endo,i} \quad (22)$$

$$\frac{d(^{212}Bi\text{-}SSTA)_{endo,i}}{dt} = \lambda_{intern} \cdot (^{212}Bi\text{-}SSTA)_{bound,i} - \lambda_{rec} \cdot (^{212}Bi\text{-}SSTA)_{endo,i} - \lambda_{sort} \cdot (^{212}Bi\text{-}SSTA)_{endo,i} + \lambda_{phy,gr}(^{212}Pb) \cdot (^{212}Pb\text{-}SSA)_{endo,i} - \lambda_{phy}(^{212}Bi) \cdot (^{212}Bi\text{-}SSTA)_{endo,i} \quad (23)$$

$$\frac{d(^{212}Bi)_{endo,i}}{dt} = \lambda_{up} \cdot (^{212}Bi)_{inters,i} - \lambda_{phy}(^{212}Bi) \cdot (^{212}Bi)_{endo,i} + \lambda_{phy,ex}(^{212}Pb) \cdot (^{212}Pb\text{-}SSTA)_{endo,i} \quad (24)$$

$$\frac{d(^{208}Tl)_{endo,i}}{dt} = \lambda_{phy,\alpha}(^{212}Bi) \cdot [(^{212}Bi\text{-}SSTA)_{endo,i} + (^{212}Bi)_{endo,i}] - \lambda_{phy}(^{208}Tl) \cdot (^{208}Tl)_{endo,i} \quad (25)$$

$$\frac{d(^{208}Pb)_{endo,i}}{dt} = \lambda_{phy,\beta}(^{212}Bi) \cdot [(^{212}Bi\text{-}SSTA)_{endo,i} + (^{212}Bi)_{endo,i}] + \lambda_{phy}(^{208}Tl) \cdot (^{208}Tl)_{endo\ i} \quad (26)$$

Differential equations for the *in vivo* alpha particle generator in the sorting compartment of SSTR2-expressing tissues:

$$\frac{d(^{212}Pb\text{-}SSTA)_{sort,i}}{dt} = \lambda_{sort} \cdot (^{212}Pb\text{-}SSTA)_{endo,i} - \lambda_{rel} \cdot (^{212}Pb\text{-}SSTA)_{sort,i} - \lambda_{phy}(^{212}Pb) \cdot (^{212}Pb\text{-}SSTA)_{sort,i} \quad (27)$$

$$\frac{d(^{212}Bi\text{-}SSTA)_{sort,i}}{dt} = \lambda_{sort} \cdot (^{212}Bi\text{-}SSTA)_{endo,i} - \lambda_{rel} \cdot (^{212}Bi\text{-}SSTA)_{sort,i} - \lambda_{phy}(^{212}Bi) \cdot (^{212}Bi\text{-}SSTA)_{sort,i} + \lambda_{phy,gr}(^{212}Pb) \cdot (^{212}Pb\text{-}SSTA)_{sort,i} \quad (28)$$

$$\frac{d(^{212}Bi)_{sort,i}}{dt} = -\lambda_{phy}(^{212}Bi) \cdot (^{212}Bi)_{sort,i} + \lambda_{phy,ex}(^{212}Pb) \cdot (^{212}Pb\text{-}SSTA)_{sort,i} \quad (29)$$

$$\frac{d(^{208}Tl)_{sort,i}}{dt} = \lambda_{phy,\alpha}(^{212}Bi) \cdot [(^{212}Bi\text{-}SSTA)_{sort,i} + (^{212}Bi)_{sort,i}] - \lambda_{phy}(^{208}Tl) \cdot (^{208}Tl)_{sort,i} \quad (30)$$

$$\frac{d(^{208}Pb)_{sort,i}}{dt} = \lambda_{phy,\beta}(^{212}Bi) \cdot [(^{212}Bi\text{-}SSTA)_{sort,i} + (^{212}Bi)_{sort,i}] + \lambda_{phy}(^{208}Tl) \cdot (^{208}Tl)_{sort,i} \quad (31)$$

Differential equations for the *in vivo* alpha particle generator in the vascular compartment of kidneys:

$$\frac{d(^{212}Pb\text{-}SSTA)_{v,K}}{dt} = FK \cdot \left(\frac{(^{212}Pb\text{-}SSTA)_{Art}}{V_{Art}} - \frac{(^{212}Pb\text{-}SSTA)_{v,K}}{V_{v,K}}\right) + \lambda_{\text{rec}} \cdot (^{212}Pb\text{-}SSTA)_{endo,K} + koff \cdot (^{212}Pb\text{-}SSTA)_{bound,K} - \frac{k_{on}}{V_{v,K}} \cdot RK \cdot (^{212}Pb\text{-}SSTA)_{v,K} - GFR \cdot \Theta_p \cdot \frac{(^{212}Pb\text{-}SSTA)_{v,K}}{V_{v,K}} - \lambda_{phy}(^{212}Pb) \cdot (^{212}Pb\text{-}SSTA)_{v,K} \quad (32)$$

$$\frac{d(^{212}Bi\text{-}SSTA)_{v,K}}{dt} = FK \cdot \left(\frac{(^{212}Bi\text{-}SSTA)_{Art}}{V_{Art}} - \frac{(^{212}Bi\text{-}SSTA)_{v,K}}{V_{v,K}}\right) + \lambda_{\text{rec}} \cdot (^{212}Bi\text{-}SSTA)_{endo,K} + koff \cdot (^{212}Bi\text{-}SSTA)_{bound,K} - \frac{k_{on}}{V_{v,K}} \cdot RK \cdot (^{212}Bi\text{-}SSTA)_{v,K} - GFR \cdot \Theta_p \cdot \frac{(^{212}Bi\text{-}SSTA)_{v,K}}{V_{v,K}} + \lambda_{phy,gr}(^{212}Pb) \cdot (^{212}Pb\text{-}SSTA)_{v,K} - \lambda_{phy}(^{212}Bi) \cdot (^{212}Bi\text{-}SSTA)_{v,K} \quad (33)$$

$$\frac{d(^{212}Bi)_{v,K}}{dt} = FK \cdot \left(\frac{(^{212}Bi)_{Art}}{V_{Art}} - \frac{(^{212}Bi)_{v,K}}{V_{v,K}}\right) - GFR \cdot \Theta_f \cdot \frac{(^{212}Bi)_{v,K}}{V_{v,K}} + (^{212}Bi\text{-}HWPP\text{-}diss) \cdot (^{212}Bi\text{-}HWPP)_{v,K} - (^{212}Bi\text{-}HWPP\text{-}ass) \cdot (^{212}Bi)_{v,K} + (^{212}Bi\text{-}RBC\text{-}diss) \cdot (^{212}Bi\text{-}RBC)_{v,K} - (^{212}Bi\text{-}RBC\text{-}ass) \cdot (^{212}Bi)_{v,K} - \lambda_{phy}(^{212}Bi) \cdot (^{212}Bi)_{v,K} + \lambda_{phy,ex}(^{212}Pb) \cdot (^{212}Pb\text{-}SSTA)_{v,K} + \lambda_{phy,ex}(^{212}Pb) \cdot (^{212}Pb\text{-}SSTA)_{bound,K} \quad (34)$$

$$\frac{d(^{212}Bi\text{-}HWPP)_{v,K}}{dt} = F_K \cdot \left(\frac{(^{212}Bi\text{-}HWPP)_{Art}}{V_{Art}} - \frac{(^{212}Bi\text{-}HWPP)_{v,K}}{V_{v,K}}\right) + (^{212}Bi\text{-}HWPP\text{-}ass) \cdot (^{212}Bi)_{v,K} - (^{212}Bi\text{-}RBC\text{-}diss) \cdot (^{212}Bi\text{-}HWPP)_{v,K} - \lambda_{phy}(^{212}Bi) \cdot (^{212}Bi\text{-}HWPP)_{v,K} \quad (35)$$

$$\frac{d(^{212}Bi\text{-}RBC)_{v,K}}{dt} = F_K \cdot \left(\frac{(^{212}Bi\text{-}RBC)_{Art}}{V_{Art}} - \frac{(^{212}Bi\text{-}RBC)_{v,K}}{V_{v,K}}\right) + (^{212}Bi\text{-}RBC\text{-}ass) \cdot (^{212}Bi)_{v,K} - (^{212}Bi\text{-}RBC\text{-}diss) \cdot (^{212}Bi\text{-}RBC)_{v,K} - \lambda_{phy}(^{212}Bi) \cdot (^{212}Bi\text{-}RBC)_{v,K} \quad (36)$$

$$\frac{d(^{208}Tl)_{v,K}}{dt} = F_K \cdot \left(\frac{(^{208}Tl)_{Art}}{V_{Art}} - \frac{(^{208}Tl)_{v,K}}{V_{v,K}}\right) + \lambda_{phy,\alpha}(^{212}Bi) \cdot [(^{212}Bi\text{-}SSTA)_{v,K} + (^{212}Bi\text{-}SSTA)_{bound,K} + (^{212}Bi)_{v,K}] - \lambda_{phy}(^{208}Tl) \cdot (^{208}Tl)_{v,K} \quad (37)$$

$$\frac{d(^{208}Pb)_{v,K}}{dt} = \lambda_{phy,\beta}(^{212}Bi) \cdot [(^{212}Bi\text{-}SSTA)_{v,K} + (^{212}Bi\text{-}SSTA)_{bound,K} + (^{212}Bi)_{v,K}] - \lambda_{phy}(^{208}Tl) \cdot (^{208}Tl)_{v,K} \quad (38)$$

Differential equations for the *in vivo* alpha particle generator for the radiolabeled SSTA-SSTR2 complexes on cell surfaces of kidneys:

$$\frac{d(^{212}Pb\text{-}SSTA)_{bound,K}}{dt} = -k_{off} \cdot (^{212}Pb\text{-}SSTA)_{bound,K} + \frac{k_{on}}{V_{v,K}} \cdot RK \cdot (^{212}Pb\text{-}SSTA)_{v,K} - \lambda_{intern} \cdot (^{212}Pb\text{-}SSTA)_{bound,k} - \lambda_{phy}(^{212}Pb) \cdot (^{212}Pb\text{-}SSTA)_{bound,K} \quad (39)$$

$$\frac{d(^{212}Bi\text{-}SSTA)_{bound,K}}{dt} = -k_{off} \cdot (^{212}Bi\text{-}SSTA)_{bound,K} + \frac{k_{on}}{V_{v,K}} \cdot RK \cdot (^{212}Bi\text{-}SSTA)_{inters,i} - \lambda_{intern} \cdot (^{212}Bi\text{-}SSTA)_{bound,k} + \lambda_{phy,gr}(^{212}Pb) \cdot (^{212}Pb\text{-}SSTA)_{bound,i} - \lambda_{phy}(^{212}Bi) \cdot (^{212}Bi\text{-}SSTA)_{bound,k}. \quad (40)$$

Differential equations for *in vivo* alpha particle generator in the endosomal compartment of kidneys:

$$\frac{d(^{212}Pb\text{-}SSTA)_{endo,K}}{dt} = \lambda_{intern} \cdot (^{212}Pb\text{-}SSTA)_{bound,K} - \lambda_{rec} \cdot (^{212}Pb\text{-}SSTA)_{endo,K} - \lambda_{sort} \cdot (^{212}Pb\text{-}SSTA)_{endo,K} - \lambda_{phy}(^{212}Pb) \cdot (^{212}Pb\text{-}SSTA)_{endo,K} \quad (41)$$

$$\frac{d(^{212}Bi\text{-}SSTA)_{endo,K}}{dt} = \lambda_{intern} \cdot (^{212}Bi\text{-}SSTA)_{bound,K} - \lambda_{rec} \cdot (^{212}Bi\text{-}SSTA)_{endo,K} - \lambda_{sort} \cdot (^{212}Bi\text{-}SSTA)_{endo,K} + \lambda_{phy}(^{212}Pb) \cdot (^{212}Pb\text{-}SSTA)_{endo,K} - \lambda_{phy}(^{212}Bi) \cdot (^{212}Bi\text{-}SSTA)_{endo,K} \quad (42)$$

$$\frac{d(^{212}Bi)_{endo,K}}{dt} = -\lambda_{phy}(^{212}Bi) \cdot (^{212}Bi)_{endo,K} + \lambda_{phy,ex}(^{212}Pb) \cdot (^{212}Pb\text{-}SSTA)_{endo,K} \tag{43}$$

$$\frac{d(^{208}Tl)_{endo,K}}{dt} = \lambda_{phy,\alpha}(^{212}Bi) \cdot [(^{212}Bi\text{-}SSTA)_{endo,K} + (^{212}Bi)_{endo,K}] - \lambda_{phy}(^{208}Tl) \cdot (^{208}Tl)_{endo,K} \tag{44}$$

$$\frac{d(^{208}Pb)_{endo,K}}{dt} = \lambda_{phy,\beta}(^{212}Bi) \cdot [(^{212}Bi\text{-}SSA)_{endo,K} + (^{212}Bi)_{endo,K}] + \lambda_{phy}(^{208}Tl) \cdot (^{208}Tl)_{endo,K} \tag{45}$$

Differential equations for *in vivo* alpha particle generator in the sorting compartment of kidneys:

$$\frac{d(^{212}Pb\text{-}SSTA)_{sort,K}}{dt} = \lambda_{\text{sort}} \cdot (^{212}Pb\text{-}SSTA)_{endo,K} - \lambda_{rel} \cdot (^{212}Pb\text{-}SSTA)_{sort,K} - \lambda_{phy}(^{212}Pb) \cdot (^{212}Pb\text{-}SSTA)_{sort,K} \tag{46}$$

$$\frac{d(^{212}Bi\text{-}SSTA)_{sort,K}}{dt} = \lambda_{\text{sort}} \cdot (^{212}Bi\text{-}SSTA)_{endo,K} - \lambda_{rel} \cdot (^{212}Bi\text{-}SSTA)_{sort,K} - \lambda_{phy}(^{212}Bi) \cdot (^{212}Bi\text{-}SSTA)_{sort,K} + \lambda_{phy,gr}(^{212}Pb) \cdot (^{212}Pb\text{-}SSTA)_{sort,K} \tag{47}$$

$$\frac{d(^{212}Bi)_{sort,K}}{dt} = -\lambda_{phy}(^{212}Bi) \cdot (^{212}Bi)_{sort,K} + \lambda_{phy,ex}(^{212}Pb) \cdot (^{212}Pb\text{-}SSTA)_{sort,K} \tag{48}$$

$$\frac{d(^{208}Tl)_{sort,K}}{dt} = \lambda_{phy,\alpha}(^{212}Bi) \cdot [(^{212}Bi\text{-}SSTA)_{sort,K} + (^{212}Bi)_{sort,K}] - \lambda_{phy}(^{208}Tl) \cdot (^{208}Tl)_{sort,K} \tag{49}$$

$$\frac{d(^{208}Pb)_{sort,K}}{dt} = \lambda_{phy,\beta}(^{212}Bi) \cdot [(^{212}Bi\text{-}SSTA)_{sort,K} + (^{212}Bi)_{sort,K}] + \lambda_{phy}(^{208}Tl) \cdot (^{208}Tl)_{sort,K} \tag{50}$$

Differential equations for *in vivo* alpha particle generator in the proximal tubule compartment of kidneys:

$$\frac{d(^{212}Pb\text{-}SSTA)_{Tub,K}}{dt} = GFR \cdot \Theta_p \cdot \frac{(^{212}Pb\text{-}SSTA)_{v,K}}{V_{v,K}} - GFR \cdot \Theta_p \cdot f_{\text{exc,p}} \cdot \frac{(^{212}Pb\text{-}SSTA)_{Tub,K}}{V_{Tub,K}} - \lambda_{endo,K} \cdot (^{212}Pb\text{-}SSTA)_{Tub,K} - \lambda_{phy}(^{212}Pb) \cdot (^{212}Pb\text{-}SSTA)_{Tub,K} \tag{51}$$

$$\frac{d(^{212}Bi\text{-}SSTA)_{Tub,K}}{dt} = GFR \cdot \Theta_p \cdot \frac{(^{212}Bi\text{-}SSTA)_{v,K}}{V_{v,K}} - GFR \cdot \Theta_p \cdot f_{\text{exc,p}} \cdot \frac{(^{212}Bi\text{-}SSTA)_{Tub,K}}{V_{Tub,K}} - \lambda_{endo,K} \cdot (^{212}Bi\text{-}SSTA)_{Tub,K} + \lambda_{phy,gr}(^{212}Pb) \cdot (^{212}Pb\text{-}SSTA)_{Tub,K} - \lambda_{phy}(^{212}Bi) \cdot (^{212}Bi\text{-}SSTA)_{Tub,K} \quad (52)$$

$$\frac{d(^{212}Bi)_{Tub,K}}{dt} = GFR \cdot \Theta_f \cdot \frac{(^{212}Bi)_{v,K}}{V_{v,K}} - GFR \cdot \Theta_f \cdot f_{\text{exc,f}} \cdot \frac{(^{212}Bi)_{Tub,K}}{V_{Tub,K}} - \lambda_{up,K} \cdot (^{212}Bi)_{Tub,K} + \lambda_{phy,ex}(^{212}Pb) \cdot (^{212}Pb\text{-}SSTA)_{Tub,K} - \lambda_{phy}(^{212}Bi) \cdot (^{212}Bi)_{Tub,K} \quad (53)$$

$$\frac{d(^{208}Tl)_{Tub,K}}{dt} = GFR \cdot \Theta_f \cdot \frac{(^{208}Tl)_{v,K}}{V_{v,K}} + \lambda_{phy,\alpha}(^{212}Bi).\,[(^{212}Bi\text{-}SSTA)_{Tub,K} + (^{212}Bi)_{Tub,K}] - \lambda_{phy}(^{208}Tl).(^{208}Tl)_{Tub,K} \quad (54)$$

$$\frac{d(^{208}Pb)_{Tub,K}}{dt} = \lambda_{phy,\beta}(^{212}Bi) \cdot [(^{212}Bi\text{-}SSTA)_{Tub,K} + (^{212}Bi)_{Tub,K}] + \lambda_{phy}(^{208}Tl) \cdot (^{208}Tl)_{Tub,K} \quad (55)$$

Differential equations for *in vivo* alpha particle generator in the renal non-specific uptake compartment of kidneys:

$$\frac{d(^{212}Pb\text{-}SSTA)_{nonsp,K}}{dt} = \lambda_{endo,K} \cdot (^{212}Pb\text{-}SSTA)_{Tub,K} - \lambda_{phy}(^{212}Pb) \cdot (^{212}Pb\text{-}SSTA)_{nonsp,K} \quad (56)$$

$$\frac{d(^{212}Bi\text{-}SSTA)_{nonsp,K}}{dt} = \lambda_{endo,K} \cdot (^{212}Bi\text{-}SSTA)_{Tub,K} + \lambda_{phy,gr}(^{212}Pb) \cdot (^{212}Pb\text{-}SSTA)_{nonsp,K} - \lambda_{phy}(^{212}Bi) \cdot (^{212}Bi\text{-}SSTA)_{nonsp,K} \quad (57)$$

$$\frac{d(^{212}Bi)_{nonsp,K}}{dt} = \lambda_{up,K} \cdot (^{212}Bi)_{Tub,K} + \lambda_{phy,ex}(^{212}Pb) \cdot (^{212}Pb\text{-}SSTA)_{nonsp,K} - \lambda_{phy}(^{212}Bi) \cdot (^{212}Bi)_{nonsp,K} \quad (58)$$

$$\frac{d(^{208}Tl)_{nonsp,K}}{dt} = \lambda_{phy,\alpha}(^{212}Bi) \cdot [(^{212}Bi\text{-}SSTA)_{nonsp,K} + (^{212}Bi)_{nonsp,K}] - \lambda_{phy}(^{208}Tl) \cdot (^{208}Tl)_{nonsp,K} \quad (58)$$

$$\frac{d(^{208}Pb)_{nonsp,K}}{dt} = \lambda_{phy,\beta}(^{212}Bi).\,[(^{212}Bi\text{-}SSTA)_{nonsp,K} + (^{212}Bi)_{nonsp,K}] + \lambda_{phy}(^{208}Tl) \cdot (^{208}Tl)_{nonsp,K} \quad (59)$$

Differential equations for *in vivo* alpha particle generator in the excretion compartment of kidneys:

$$\frac{d({}^{212}Pb\text{-}SSTA)_{exc,K}}{dt} = GFR \cdot \Theta_p \cdot f_{\text{exc,p}} \cdot \frac{({}^{212}Pb\text{-}SSTA)_{Tub,K}}{V_{Tub,K}} - \lambda_{phy}({}^{212}Pb) \cdot ({}^{212}Pb\text{-}SSTA)_{exc,K} \tag{60}$$

$$\frac{d({}^{212}Bi\text{-}SSA)_{exc,K}}{dt} = GFR \cdot \Theta_p \cdot f_{\text{exc,p}} \cdot \frac{({}^{212}Bi\text{-}SSTA)_{Tub,K}}{V_{Tub,K}} + \lambda_{phy,gr}({}^{212}Pb) \cdot ({}^{212}Pb\text{-}SSTA)_{exc,K} - \lambda_{phy}({}^{212}Bi) \cdot ({}^{212}Bi\text{-}SSTA)_{exc,K} \tag{61}$$

$$\frac{d({}^{212}Bi)_{exc,K}}{dt} = GFR \cdot \Theta_f \cdot f_{\text{exc,f}} \cdot \frac{({}^{212}Bi)_{Tub,K}}{V_{Tub,K}} + \lambda_{phy,ex}({}^{212}Pb) \cdot ({}^{212}Pb\text{-}SSTA)_{exc,K} - \lambda_{phy}({}^{212}Bi) \cdot ({}^{212}Bi\text{-}SSTA)_{exc.K} \tag{62}$$

$$\frac{d({}^{208}Tl)_{exc,K}}{dt} = GFR \cdot \Theta_f \cdot f_{\text{exc,f}} \cdot \frac{({}^{212}Bi)_{Tub,K}}{V_{Tub,K}} + \lambda_{phy,\alpha}({}^{212}Bi) \cdot [({}^{212}Bi\text{-}SSTA)_{exc,K} + ({}^{212}Bi)_{exc,K}] - \lambda_{phy}({}^{208}Tl) \cdot ({}^{208}Tl)_{exc.K} \tag{63}$$

$$\frac{d({}^{208}Pb)_{exc,K}}{dt} = \lambda_{phy,\beta}({}^{212}Bi) \cdot [({}^{212}Bi\text{-}SSA)_{exc,K} + ({}^{212}Bi)_{exc,K}] + \lambda_{phy}({}^{208}Tl) \cdot ({}^{208}Tl)_{exc,K} \tag{64}$$

PBPK model parameters

Table S1. [^{212}Pb]Pb-SSTA-PBPK model parameters.

**[^{212}Pb]Pb-DOTAMTATE Specific Parameters**				
	**Variable**	**Value**	**Unit**	**Source**
k_{on}	Association rate	k_{off} / KD	L·nmol^{-1}·min^{-1}	[1]
k_{off}	Dissociation rate	0.04	min^{-1}	[1]
KD	Dissociation constant	1	nmol·L^{-1}	[1]
λ_{phy} (^{212}Pb)	Physical decay rate of ^{212}Pb	0.00109	min^{-1}	[2]
f	Dissociation fraction of Bi-DOTAMTATE complex	0.16	–	[3]
$\lambda_{phy,ex}$(^{212}Pb)	Beta physical decay rate of ^{212}Pb to the excited states of ^{212}Bi	$f \cdot \lambda_{phy}$(^{212}Pb)	min^{-1}	[3]
$\lambda_{phy,gr}$(^{212}Pb)	Beta physical decay rate of ^{212}Pb directly to the ground states	(1-f)·λ_{phy}(^{212}Pb)	min^{-1}	[3]
$\lambda_{phy,\beta}$ (^{212}Bi)	Beta physical decay rate of ^{212}Bi	0.00732	min^{-1}	[2]
$\lambda_{phy,\alpha}$ (^{212}Bi)	Alpha physical decay rate of ^{212}Bi	0.00412	min^{-1}	[2]
$\lambda_{phy,\beta}$ (^{208}Tl)	Beta physical decay rate of ^{208}Tl	0.22360	min^{-1}	[2]
$E_{Pb,\beta}$	Emitted energy by beta decay of ^{212}Pb	0.57	MeV	[2]
$E_{Bi,\beta}$	Emitted energy by beta decay of ^{212}Bi	2.2	MeV	[2]
$E_{Bi,\alpha}$	Emitted energy by alpha decay of ^{212}Bi	6.1	MeV	[2]
$E_{Po,\alpha}$	Emitted energy by alpha decay of ^{212}Po	8.8	MeV	[2]
$E_{Tl,\beta}$	Emitted energy by beta decay of ^{208}Tl	1.8	MeV	[2]

**Mice Specific Parameters**				
BW	Body weight of a mouse	0.03	kg	
H	Hematocrit	0.45	unity	[4]
V_P	Volume of total body serum	0.059·BW	L	[5]
CO	Cardiac output	$CO=0.275\cdot(BW(kg))^{0.75}$	$L\cdot min^{-1}$	[6]
**Tumor**				
$V_{T,total}$	Total volume of tumor	$300\cdot10^{-6}$	L	[7]
$V_{int,T}$	Interstitial space of tumor	$v_{t,int}\cdot V_{T,total}$	L	
$V_{v,T}$	Vascular (serum) space of tumor	$v_{t,v}\cdot V_{T,total}\cdot(1\text{-}H)$	L	
$V_{vRBC,T}$	Vascular (RBC) space of tumor	$v_{t,v}\cdot V_{T,total}\cdot(H/(1\text{-}H))$	L	
$v_{t,int}$	Fraction of the interstitial space to the total tumor volume	0.4	unity	[1]
$v_{t,v}$	Fraction of the vascular space to the total tumor volume	0.1	unity	[1]
F_T	Serum flow to tumor	$f_T\cdot V_{T,total}$	$L\cdot min^{-1}$	
f_T	Serum flow density to tumor	fitted	$mL\cdot min^{-1}\cdot g^{-1}$	
PS_T	Permeability surface area product of peptides in tumor	$k_T\cdot V_{T,\ total}$	$mL\cdot min^{-1}$	
k_T	Permeability surface area product of peptides per unit mass of tumor (scaled for molecule size of DOTAM-TATE)	0.31	$mL\cdot min^{-1}\cdot g^{-1}$	[1]
$[R_T]$	sst2 receptor density of tumor	fitted	$nmol\cdot L^{-1}$	
R_T	sst2 receptor number of tumor	$[R_T]\cdot V_{T,total}$	nmol	
$\lambda_{T,release}$	Release rate of tumor	0.0 (Preliminary fit)	min^{-1}	
**Kidneys**				
GFR	Glomerular filtration rate for mice	0.0001	$L\cdot min^{-1}$	
Θ_p	Ratio of sieving coefficient for peptides	0.97	unity	[8]
$Filtration_p$	Filtration of peptides	$GFR\cdot\Theta_p$	$L\cdot min^{-1}$	
$f_{exc,p}$	Excretion fraction of peptides	0.98	Unity	
$Excretion_p$	Excretion of peptides	$Filtration_p\cdot f_{exc,p}$	L	
Θ_f	Ratio of sieving coefficient for free radionuclides	1	unity	[9]
$Filtration_f$	Filtration of free radionuclides	$GFR\cdot\Theta_f$	$L\cdot min^{-1}$	
$f_{exc,f}$	Excretion fraction of free radionuclides	0.0	Unity	[10]
$Excretion_f$	Excretion of free radionuclides	$Filtration_f\cdot f_{exc,f}$	L	
**Other Organs**				
$V_{i,total}$	Total volume of tissue i		L	
$V_{K,total}$	Total volume of kidneys	0.019·BW	L	[11]
$V_{L,total}$	Total volume of liver	0.069·BW	L	[11]
$V_{Sp,total}$	Total volume of spleen	0.005·BW	L	[11]
$V_{Panc,total}$	Total volume of pancreas	0.003·BW	L	[11]
$V_{GI,total}$	Total volume of gastrointestinal tract (GI)	0.037·BW	L	[11]
$V_{Ad,total}$	Total volume of adrenal gland	0.0005·BW	L	[6]
$V_{Lun,total}$	Total volume of lung	0.007·BW	L	[11]
$V_{Mus,total}$	Total volume of muscle	0.403·BW	L	[11]

$V_{Skin,total}$	Total volume of skin	$0.188 \cdot BW$	L	[11]
$V_{Hrt,total}$	Total volume of heart	$0.005 \cdot BW$	L	[11]
$V_{Br,total}$	Total volume of brain	$0.017 \cdot BW$	L	[11]
$V_{Bone,total}$	Total volume of bone	$(0.054/\varrho_{Bone}) \cdot BW$	L	[6]
ϱ_{Bone}	Marrow-free bone density	1.92	$g \cdot cm^{-3}$	[6]
$V_{Fat,total}$	Total volume of fat	$(0.071/\varrho_{Fat}) \cdot BW$	l	[11]
ϱ_{Fat}	Fat density	0.92	$g \cdot cm^{-3}$	[6]
V_{BW}	Volume of total body based on BW except tumor	1 ml ≙ 1 g (for all body organs except bone and fat)	L	
$V_{RB,total}$	Total volume of remainder of body	$V_{BW} - \sum_i V_{i,total}$	L	
$V_{v,i}$	Vascular (serum) volume of tissue i	$V_{i,total} \cdot v_{v,i}$	L	
$v_{v,i}$	Fraction of the vascular serum volume to total tissue volume of an average mouse	$V_{i,v}/ V_{i,total}$	unity	
$V_{v,K}$	Vascular (serum) volume of kidneys	$V_{K,total} \cdot 0.055$	L	[11]
$V_{v,L}$	Vascular (serum) volume of liver	$V_{L,total} \cdot 0.085$	L	[11]
$V_{v,Sp}$	Vascular (serum) volume of spleen	$V_{Sp,total} \cdot 0.121$	L	[11]
$V_{v,Panc}$	Vascular (serum) volume of pancreas	$V_{Panc,total} \cdot 0.055$	L	[11]
$V_{v,GI}$	Vascular (serum) volume of GI	$V_{GI,total} \cdot 0.032$	L	[11]
$V_{v,Ad}$	Vascular (serum) volume of Adrenal	$V_{Ad,total} \cdot 0.03 \cdot (1-H)$	L	[6]
$V_{v,Lun}$	Vascular (serum) volume of lung	$V_{Lun,total} \cdot 0.145$	L	[11]
$V_{v,Mus}$	Vascular (serum) volume of muscles	$V_{Mus,total} \cdot 0.022$	L	[11]
$V_{v,Skin}$	Vascular (serum) volume of skin	$V_{Skin,total} \cdot 0.037$	L	[11]
$V_{v,Hrt}$	Vascular (serum) volume of heart	$V_{Hrt,total} \cdot 0.038$	L	[11]
$V_{v,Br}$	Vascular (serum) volume of brain	$V_{Br,total} \cdot 0.022$	L	[11]
$V_{v,Bone}$	Vascular (serum) volume of bone	$V_{Bone,total} \cdot 0.11 \cdot (1-H)$	L	[6]
$V_{v,Fat}$	Vascular (serum) volume of fat	$V_{Fat,total} \cdot 0.011$	L	[11]
V_{Art}	Arterial serum plus ½ serum content of heart	$0.06 \cdot V_P + 0.045 \cdot V_P$	L	[12]
V_{Vein}	Venous serum plus ½ serum content of heart	$0.18 \cdot V_P + 0.045 \cdot V_P$	L	[12]
$V_{v,RB}$	Serum volume of remainder of body	$V_P - \sum_i V_{i,v}$	L	
V_{RBC}	Total volume of red blood cells (RBC)	$BW \cdot 0.0276$	L	[11]
$V_{vRBC,i}$	Vascular RBC volume of tissue i	$f_{sc} \cdot V_{i,total} \cdot v_{vRBC,i}$	L	
f_{sc}	Scaling factor	0.86	Unity	
$v_{vRBC,i}$	Fraction of the vascular (RBC) volume to total tissue volume of an average mouse	$V_{v,RBC,i}/ V_{i,total}$	L	
$V_{vRBC,K}$	Vascular (RBC) volume of kidneys	$f_{sc} \cdot V_{K,total} \cdot 0.045$	L	[11]
$V_{vRBC,L}$	Vascular (RBC) volume of liver	$f_{sc} \cdot V_{L,total} \cdot 0.069$	L	[11]
$V_{vRBC,Sp}$	Vascular (RBC) volume of spleen	$f_{sc} \cdot V_{Sp,total} \cdot 0.099$	L	[11]
$V_{vRBC,Panc}$	Vascular (RBC) volume of pancreas	$f_{sc} \cdot V_{Panc,total} \cdot 0.045$	L	[11]
$V_{vRBC,GI}$	Vascular (RBC) volume of GI	$f_{sc} \cdot V_{GI,total} \cdot 0.01$	L	[11]
$V_{vRBC,Ad}$	Vascular (RBC) volume of Adrenal	$f_{sc} \cdot V_{Ad,total} \cdot 0.03 \cdot H$	L	[11]
$V_{vRBC,Lun}$	Vascular (RBC) volume of lung	$f_{sc} \cdot V_{Lun,total} \cdot 0.118$	L	[11]
$V_{vRBC,Mus}$	Vascular (RBC) volume of muscles	$f_{sc} \cdot V_{Mus,total} \cdot 0.018$	L	[11]
$V_{vRBC,Skin}$	Vascular (RBC) volume of skin	$f_{sc} \cdot V_{Skin,total} \cdot 0.031$	L	[11]

$V_{vRBC,Hrt}$	Vascular (RBC) volume of heart	$f_{sc}\cdot V_{Hrt,total}\cdot 0.032$	L	[11]
$V_{vRBC,Br}$	Vascular (RBC) volume of brain	$f_{sc}\cdot V_{Br,total}\cdot 0.018$	L	[11]
$V_{vRBC,Bone}$	Vascular (RBC) volume of bone	$f_{sc}\cdot V_{Bone,total}\cdot 0.01\cdot H$	L	[6]
$V_{vRBC,Fat}$	Vascular (RBC) volume of fat	$f_{sc}\cdot V_{Fat,total}\cdot 0.008$	L	[11]
$V_{Art,RBC}$	Arterial RBC plus ½ RBC content of heart	$f_{sc}\cdot(0.06\cdot V_P+0.045\cdot V_P)$	L	[1]
$V_{Vein,RBC}$	Venous RBC plus ½ RBC content of heart	$f_{sc}\cdot(0.18\cdot V_P+0.045\cdot V_P)$	L	[1]
$V_{vRBC,RB}$*	RBC volume of remainder of body	$f_{sc}\cdot V_{vRBC,Ad}$	L	
$V_{inters,i}$	Interstitial volume of tissue i	$V_{i,total}\cdot v_{i,inters}$	L	
$v_{inters,\ i}$	Fraction of interstitial volume to total tissue volume of an average mouse	$V_{inters,i}/\ V_{i,total}$	unity	
$V_{inters,L}$	Interstitial volume of liver	$V_{L,total}\cdot 0.2$	L	[11]
$V_{inters,Sp}$	Interstitial volume of spleen	$V_{Sp,total}\cdot 0.2$	L	[11]
$V_{inters,Panc}$	Interstitial volume of pancreas	$V_{Panc,total}\cdot 0.17$	L	[11]
$V_{inters,GI}$	Interstitial volume of GI	$V_{GI,total}\cdot 0.35$	L	[11]
$V_{inters,Ad}$	Interstitial volume of Adrenal	$V_{Ad,total}\cdot 0.2$	L	[1]
$V_{inters,Lun}$	Interstitial volume of lung	$V_{Lun,total}\cdot 0.19$	L	[11]
$V_{inters,Mus}$	Interstitial volume of muscle	$V_{Mus,total}\cdot 0.13$	L	[11]
$V_{inters,Skin}$	Interstitial volume of skin	$V_{Skin,total}\cdot 0.33$	L	[11]
$V_{inters,Hrt}$	Interstitial volume of heart	$V_{Hrt,total}\cdot 0.14$	L	[11]
$V_{inters,Bone}$	Interstitial volume of bone	$V_{Bon,total}\cdot 0.19$	L	[11]
$V_{inters,Fat}$	Interstitial volume of fat	$V_{Fat,total}\cdot 0.17$	L	[11]
$V_{inters,RB}$	Interstitial volume of remainder of body	$V_{RB,total}\cdot 0.17$	L	[11]
$V_{K\_Prox\_Tub}$	Volume of kidneys proximal tubules	0.0001449	L	[13,14]
F_total_blood	Total blood flow rate	CO	$L\cdot min^{-1}$	
F_total_RBC	Total RBC flow rate	H·CO	$L\cdot min^{-1}$	
F_total_Serum	Total serum flow rate	(1-H)·CO	$L\cdot min^{-1}$	
F_i	Flow rate of blood to tissue i	$CO\cdot f_i$	$L\cdot min^{-1}$	
$F_{i,RBC}$	Flow rate of RBC to tissue i	$H\cdot CO\cdot f_i$	$L\cdot min^{-1}$	
$F_{i,p}$	Flow rate of serum to tissue i	$(1-H)\cdot CO\cdot f_i$	$L\cdot min^{-1}$	
f_i	Fraction of flow rate to tissue i to F_total_blood of an average mouse	F_i/F_total_blood	unity	
f_K	Fraction of Kidney blood flow rate to F_total_blood	0.184	unity	[11]
f_L	Fraction of liver blood flow rate to F_total_blood	0.028	unity	[11]
f_{Sp}	Fraction of spleen blood flow rate to F_total_blood	0.022	unity	[11]
f_{Panc}	Fraction of pancreas blood flow rate to F_total_blood	0.017	unity	[11]
f_{GI}	Fraction of GI blood flow rate to F_total_blood	0.135	unity	[11]
f_{Ad}	Fraction of Adrenal blood flow rate to F_total_blood	0.0027	unity	[15]
f_{Lun}	Fraction of lung blood flow rate to F_total_blood	1	unity	[11]
f_{Mus}	Fraction of muscle blood flow rate to F_total_blood	0.231	unity	[11]

f_{Skin}	Fraction of skin blood flow rate to F_total_blood	0.075	unity	[11]
f_{Hrt}	Fraction of heart blood flow rate to F_total_blood	0.098	unity	[11]
f_{Bone}	Fraction of bone blood flow rate to F_total_blood	0.041	unity	[11]
f_{Fat}	Fraction of fat blood flow rate to F_total_blood	0.036	unity	[11]
f_{RB}	Fraction of RB blood flow rate to F_total_blood	f_{Ad}	$L \cdot min^{-1}$	
	**[212Pb]Pb-DOTAMTATE and Mice Relevent Parameters**			
$PS_{i,p}$	Permeability surface area product of peptides for tissue i	$k_{i,p} \cdot V_{i,total}$	$mL \cdot min^{-1}$	
$k_{i,p}$	Permeability surface area product of peptides per unit mass of tissue i		$mL \cdot min^{-1} \cdot g^{-1}$	
$k_{Mus,p}$	Permeability surface area product of peptide per unit mass of muscle	0.02	$mL \cdot min^{-1} \cdot g^{-1}$	
$k_{L,p}$	Permeability surface area product of peptide per unit mass of liver	$k_{Mus,p} \cdot 500$	$mL \cdot min^{-1} \cdot g^{-1}$	[16]
$k_{Sp,p}$	Permeability surface area product of peptide per unit mass of spleen	$k_{Mus,p} \cdot 500$	$mL \cdot min^{-1} \cdot g^{-1}$	[16]
$k_{Panc,p}$	Permeability surface area product of peptide per unit mass of pancreas	$k_{Mus,p} \cdot 100$	$mL \cdot min^{-1} \cdot g^{-1}$	[16]
$k_{GI,p}$	Permeability surface area product of peptide per unit mass of GI	$k_{Mus,p}$	$mL \cdot min^{-1} \cdot g^{-1}$	[16]
$k_{Ad,p}$	Permeability surface area product of peptide per unit mass of adrenal	$k_{Mus,p} \cdot 100$	$mL \cdot min^{-1} \cdot g^{-1}$	[16]
$k_{Lun,p}$	Permeability surface area product of peptide per unit mass of lung	$k_{Mus,p} \cdot 100$	$mL \cdot min^{-1} \cdot g^{-1}$	[16]
$k_{Skin,p}$	Permeability surface area product of peptide per unit mass of skin	$k_{Mus,p}$	$mL \cdot min^{-1} \cdot g^{-1}$	[16]
$k_{Hrt,p}$	Permeability surface area product of peptide per unit mass of heart	$k_{Mus,p}$	$mL \cdot min^{-1} \cdot g^{-1}$	[16]
$k_{Bone,p}$	Permeability surface area product of peptide per unit mass of bone	$k_{Mus,p}$	$mL \cdot min^{-1} \cdot g^{-1}$	[16]
$k_{Fat,p}$	Permeability surface area product of peptide per unit mass of fat	$k_{Mus,p}$	$mL \cdot min^{-1} \cdot g^{-1}$	[16]
$k_{RB,p}$	Permeability surface area product of peptide per unit mass of remainder of body	$k_{Mus,p}$	$mL \cdot min^{-1} \cdot g^{-1}$	[16]
$PS_{i,f}$	Permeability surface area product of free radionuclides for tissue i	$k_{i,f} \cdot V_{i,total}$	$mL \cdot min^{-1}$	
$k_{i,f}$	Permeability surface area product of free radionuclides per unit mass of tissue i		$mL \cdot min^{-1} \cdot g^{-1}$	
$k_{Mus,f}$	Permeability surface area product of free radionuclides per unit mass of muscle	0.22	$mL \cdot min^{-1} \cdot g^{-1}$	[10]

$k_{L,f}$	Permeability surface area product of free radionuclides per unit mass of liver	$k_{Mus,f}$·500	mL·min^{-1}·g^{-1}	[16]
$k_{Sp,f}$	Permeability surface area product of free radionuclides per unit mass of spleen	$k_{Mus,f}$·500	mL·min^{-1}·g^{-1}	[16]
$k_{Panc,f}$	Permeability surface area product per unit mass of pancreas	$k_{Mus,f}$·100	mL·min^{-1}·g^{-1}	[16]
$K_{GI,f}$	Permeability surface area product of free radionuclides per unit mass of GI	$k_{Mus,f}$	mL·min^{-1}·g^{-1}	[16]
$k_{Ad,f}$	Permeability surface area product of free radionuclides per unit mass of adrenal	$k_{Mus,f}$·100	mL·min^{-1}·g^{-1}	[16]
$k_{Lun,f}$	Permeability surface area product of free radionuclides per unit mass of lung	$k_{Mus,f}$·100	mL·min^{-1}·g^{-1}	[16]
$k_{Skin,f}$	Permeability surface area product of free radionuclides per unit mass of skin	$k_{Mus,f}$	mL·min^{-1}·g^{-1}	[16]
$k_{Hrt,f}$	Permeability surface area product of free radionuclides per unit mass of heart	$k_{Mus,f}$	mL·min^{-1}·g^{-1}	[16]
$k_{Bone,f}$	Permeability surface area product of free radionuclides per unit mass of bone	$k_{Mus,f}$	mL·min^{-1}·g^{-1}	[16]
$k_{Fat,f}$	Permeability surface area product of free radionuclides per unit mass of fat	$k_{Mus,f}$	mL·min^{-1}·g^{-1}	[16]
$k_{RB,Bi}$	Permeability surface area product of free radionuclides per unit mass of remainder of body	$k_{Mus,f}$	mL·min^{-1}·g^{-1}	[16]
λ_{intern}	Internalization rate of peptides	0.17	min^{-1}	[17]
λ_{rec}	Recycling rate of peptides	0.05	min^{-1}	[18]
λ_{sort}	Sorting rate of peptides	fitted	min^{-1}	
$\lambda_{rel,NT}$	Release rate in normal tissues	0.0 (Preliminary fit)	min^{-1}	
$\lambda_{rel,Pan}$	Release rate in pancreas	fitted	min^{-1}	
$\lambda_{rel,k}$	Release rate in kidneys	fitted	min^{-1}	
$\lambda_{rel,T}$	Release rate in Tumor	0.0 (Preliminary fit)	min^{-1}	
$\lambda_{up,liv}$	The uptake rate of free ^{212}Bi by liver cells	0.86	min^{-1}	[10]
$\lambda_{up,GI}$	The uptake rate of free ^{212}Bi by GI cells	0.27	min^{-1}	[10]
$\lambda_{up,Sp}$	The uptake rate of free ^{212}Bi by spleen cells	$\lambda_{up,GI}$	min^{-1}	[10]
$\lambda_{up,Panc}$	The uptake rate of free ^{212}Bi by pancreatic cells	$\lambda_{up,GI}$	min^{-1}	[10]
$\lambda_{up,Bone}$	The uptake rate of free ^{212}Bi by Bone cells	3.85	min^{-1}	[10]
$\lambda_{up,Skin}$	The uptake rate of free ^{212}Bi by Skin cells	0.04	min^{-1}	[10]
$\lambda_{up,Mus}$	The uptake rate of ^{212}Bi by Muscle cells	0.007	min^{-1}	[10]

$\lambda_{up,Fat}$	The uptake of ^{212}Bi by Fat cells	$\lambda_{up,Mus}$	min^{-1}	[10]
$\lambda_{up,Lung}$	The uptake of ^{212}Bi by Lung cells	$\lambda_{up,GI}$	min^{-1}	[10]
$\lambda_{up,Hrt}$	The uptake of ^{212}Bi by Heart cells	$\lambda_{up,Mus}$	min^{-1}	[10]
$\lambda_{up,K}$	The uptake of ^{212}Bi by epithelial cells of proximal tubules	1	min^{-1}	[10]
$\lambda_{up,RB}$	The uptake of ^{212}Bi by remainder of body	$\lambda_{up,Mus}$	min^{-1}	[10]
$\lambda_{Endo,k}$	Renal non-specific uptake	0.03	min^{-1}	
Bi-RBC-ass	First order association rate of free ^{212}Bi with RBC	0.01	min^{-1}	[10]
Bi-HWPP-ass	First order association rate of free ^{212}Bi with high molecular weight plasma proteins	0.20	min^{-1}	[10]
Bi-RBC-diss	First order dissociation rate of Bi-RBC complex	0.017	min^{-1}	[10]
Bi-HWPP-diss	First order dissociation rate of Bi-HWPP complex	0.20	min^{-1}	[10]
$[R_i]$	sst2 receptor density of in tissue i	fitted	nmol·L^{-1}	
R_i	sst2 receptor amount in tissue i	$[R_i]\cdot V_{i,total}$	nmol	
$[R_k]$	sst2 receptor density of in Kidneys	fitted	nmol·L^{-1}	
$[R_{pan}]$	sst2 receptor density of in pancreas	fitted	nmol·L^{-1}	
$[R_{Sp}]$	sst2 receptor density of in spleen	fitted	nmol·L^{-1}	
$[R_{Liv}]$	sst2 receptor density of in liver	fitted	nmol·L^{-1}	
$[R_{Lun}]$	sst2 receptor density of in lung	fitted	nmol·L^{-1}	
$[R_{Ad}]$	sst2 receptor density of in adrenal gland	$0.4\cdot[R_{pan}]$	nmol·L^{-1}	[19]
$[R_{GI}]$	sst2 receptor density of in GI	$0.75\cdot[R_{pan}]$	nmol·L^{-1}	[19]

References

1. Kletting, P.; Kull, T.; Maaß, C.; Malik, N.; Luster, M.; Beer, A.J.; Glatting, G. Optimized peptide amount and activity for ^{90}Y-Labeled DOTATATE therapy. *J. Nucl. Med.* **2016**, *57*, 503-508, doi:10.2967/jnumed.115.164699.
2. Schupp, G.; Daniel, H.; Eakins, G.W.; Jensen, E.N. Transition Intensities in the Tl^{208} Beta Decay, the Bi^{212} ->Po^{212} Decay Scheme, and the Bi^{212} Branching Ratio. *Phys Rev* **1960**, *120*, 189-198, doi:10.1103/PhysRev.120.189.
3. Zaid, N.R.R.; Kletting, P.; Beer, A.J.; Rozgaja Stallons, T.A.; Torgue, J.J.; Glatting, G. Mathematical modeling of *in vivo* alpha particle generators and chelator stability. *Cancer Biother Radiopharm* **epub 21.01.2021**.
4. Davies, B.; Morris, T. Physiological Parameters in Laboratory Animals and Humans. *Pharm Res* **1993**, *10*, 1093-1095, doi:10.1023/A:1018943613122.
5. Riches, A.C.; Sharp, J.G.; Thomas, D.B.; Smith, S.V. Blood volume determination in the mouse. *The Journal of physiology* **1973**, *228*, 279-284, doi:10.1113/jphysiol.1973.sp010086.
6. Brown, R.P.; Delp, M.D.; Lindstedt, S.L.; Rhomberg, L.R.; Beliles, R.P. Physiological parameter values for physiologically based pharmacokinetic models. *Toxicol. Ind. Health* **1997**, *13*, 407-484.
7. Stallons, T.A.R.; Saidi, A.; Tworowska, I.; Delpassand, E.S.; Torgue, J.J. Preclinical Investigation of ^{212}Pb-DOTAMTATE for Peptide Receptor Radionuclide Therapy in a Neuroendocrine Tumor Model. *Mol Cancer Ther* **2019**, *18*, 1012-1021, doi:10.1158/1535-7163.mct-18-1103.
8. Schmidt, M.M.; Wittrup, K.D. A modeling analysis of the effects of molecular size and binding affinity on tumor targeting. *Mol Cancer Ther* **2009**, *8*, 2861-2871, doi:10.1158/1535-7163.MCT-09-0195.

9. Rabkin, R.; Dahl, D.C. Renal Uptake and Disposal of Proteins and Peptides. In *Biological Barriers to Protein Delivery*, Andus, K.L., Raub, T.L., Eds.; Pharmaceutical Biotechnology; Springer Science+Business Media, LLC: New York, 1993; Volume 4, pp. 299-338.
10. Zaid, N.R.R.; Kletting, P.; Winter, G.; Beer, A.J.; Glatting, G. A Whole-Body Physiologically Based Pharmacokinetic Model for Alpha Particle Emitting Bismuth in Rats. *Cancer Biother Radiopharm* **epub 29.06.21**, doi:10.1089/cbr.2021.0028.
11. Shah, D.K.; Betts, A.M. Towards a platform PBPK model to characterize the plasma and tissue disposition of monoclonal antibodies in preclinical species and human. *J Pharmacokinet Pharmacodyn* **2012**, *39*, 67-86, doi:10.1007/s10928-011-9232-2.
12. Leggett, R.W.; Williams, L.R. A proposed blood circulation model for reference man. *Health Phys* **1995**, *69*, 187-201.
13. Delaney, M.A.; Kowalewska, J.; Treuting, P.M. 16 - Urinary System. In *Comparative Anatomy and Histology (Second Edition)*, Treuting, P.M., Dintzis, S.M., Montine, K.S., Eds.; Academic Press: San Diego, 2018; pp. 275-301.
14. Letts, R.F.R.; Zhai, X.-Y.; Bhikha, C.; Grann, B.L.; Blom, N.B.; Thomsen, J.S.; Rubin, D.M.; Christensen, E.I.; Andreasen, A. Nephron morphometry in mice and rats using tomographic microscopy. *Am J Physiol Renal Physiol* **2017**, *312*, F210-F229, doi:10.1152/ajprenal.00207.2016.
15. Sparrow, R.A.; Coupland, R.E. Blood flow to the adrenal gland of the rat: its distribution between the cortex and the medulla before and after haemorrhage. *J Anat* **1987**, *155*, 51-61.
16. Groothuis, D.R. The blood-brain and blood-tumor barriers: a review of strategies for increasing drug delivery. *Neuro Oncol* **2000**, *2*, 45-59.
17. Hipkin, R.W.; Friedman, J.; Clark, R.B.; Eppler, C.M.; Schonbrunn, A. Agonist-induced desensitization, internalization, and phosphorylation of the sst2A somatostatin receptor. *J Biol Chem* **1997**, *272*, 13869-13876, doi:10.1074/jbc.272.21.13869.
18. Koenig, J.A.; Kaur, R.; Dodgeon, I.; Edwardson, J.M.; Humphrey, P.P.A. Fates of endocytosed somatostatin sst2 receptors and associated agonists. *Biochem J* **1998**, *336*, 291-298, doi:10.1042/bj3360291.
19. Chan, H.S.; Konijnenberg, M.W.; Daniels, T.; Nysus, M.; Makvandi, M.; de Blois, E.; Breeman, W.A.; Atcher, R.W.; de Jong, M.; Norenberg, J.P. Improved safety and efficacy of ^{213}Bi-DOTATATE-targeted alpha therapy of somatostatin receptor-expressing neuroendocrine tumors in mice pre-treated with L-lysine. *EJNMMI Res* **2016**, *6*, 83, doi:10.1186/s13550-016-0240-5.
20. Begum, N.J.; Thieme, A.; Eberhardt, N.; Tauber, R.; D'Alessandria, C.; Beer, A.J.; Glatting, G.; Eiber, M.; Kletting, P. The effect of total tumor volume on the biologically effective dose of tumor and kidneys for ^{177}Lu-labelled PSMA peptides. *J Nucl Med* **2018**, *6*, 929-933, doi:10.2967/jnumed.117.203505.

Suplement B: Biokinetic data for fitting

Biokinetic data of [^{212}Pb]Pb-DOTAMTATE reported by Stallon et al. [7] used for fitting the model parameters

Table S2. Non-decay corrected %ID/g for [^{212}Pb]Pb-DOTAMTATE in different tissues of unanesthetized AR42J-bearing mice at different time points.

Time (min)	Kidneys	Liver	Spleen	Pancreas	Tumor	Lung
60	18.7	1.4	3.8	31	21	7.9
240	12.3	0.7	2.3	11.6	17.3	5
1440	2.1	0.1	0.5	1	5.2	1.3

Suplement C: Therapeutic ratios of all tissues using the ^{212}Pb-SSTA-PBPK model

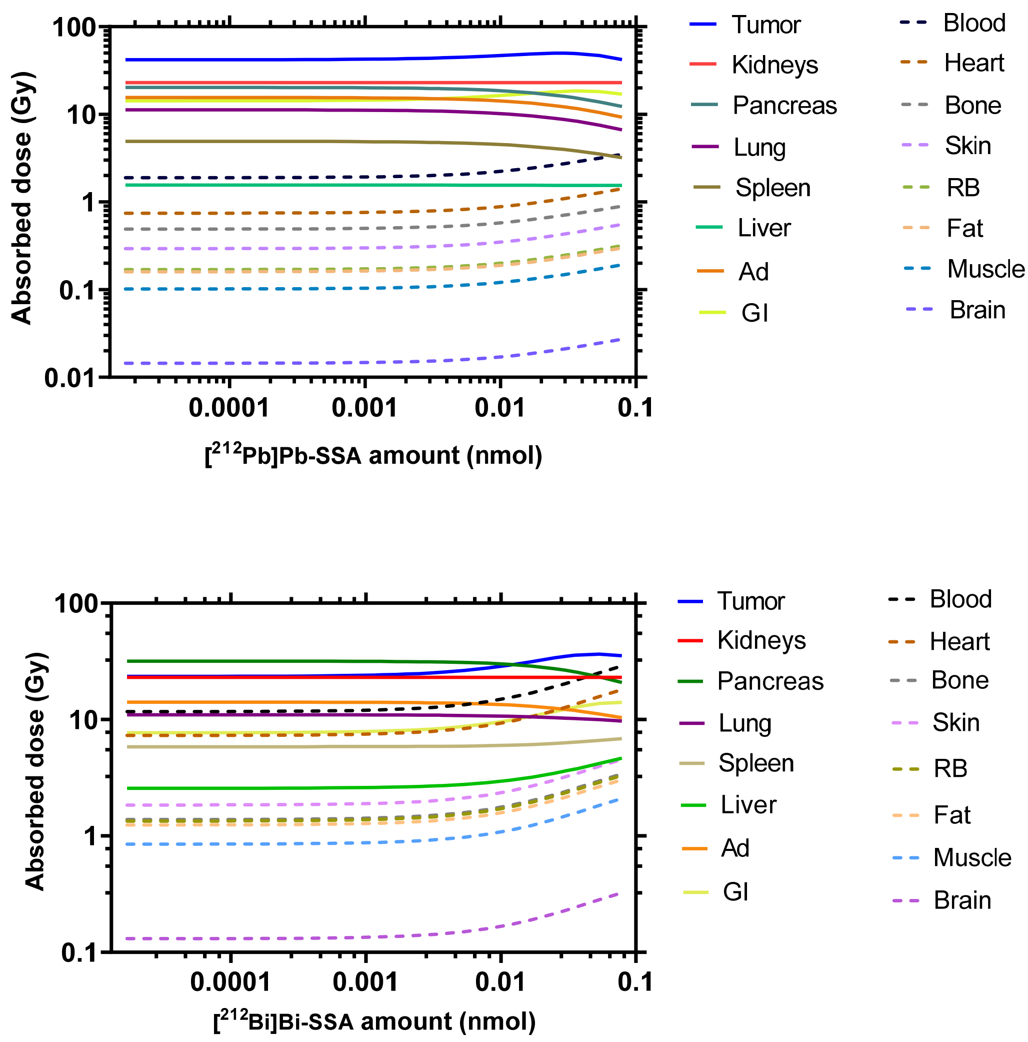

Figure S1. Comparison of the therapeutic potency of the *in vivo* alpha generator [^{212}Pb]Pb-SSTA (upper panel) and [^{212}Bi]Bi-SSTA (lower panel). The absorbed doses in SSTR2-expressing tissues (solid lines) and non-SSTR2-expressing tissues (dotted lines) were calculated for different injected amounts, with activity chosen such that kidneys always receive a dose of 23 Gy. The logarithmic presentation of the absorbed doses shows that the absorbed doses in non-SSTR2-expressing tissues in both figures are low and follow the same patterns.

Supplement D: Sensitivity Analyses

Sensitivity analyses were performed using a feature of the SAAM II modeling software that produces sensitivity plots of different model results for all fitted parameters listed in table 1. The time course of the injected dose per gram (ID/g) of tumor and organs at risk, i.e. kidneys and pancreas were investigated using a plausible range for the fitted input parameters (mean ± 0, 1, 2 and 3 SD). The generated sensitivity plots are shown below in Figures S2, S3 and S4.

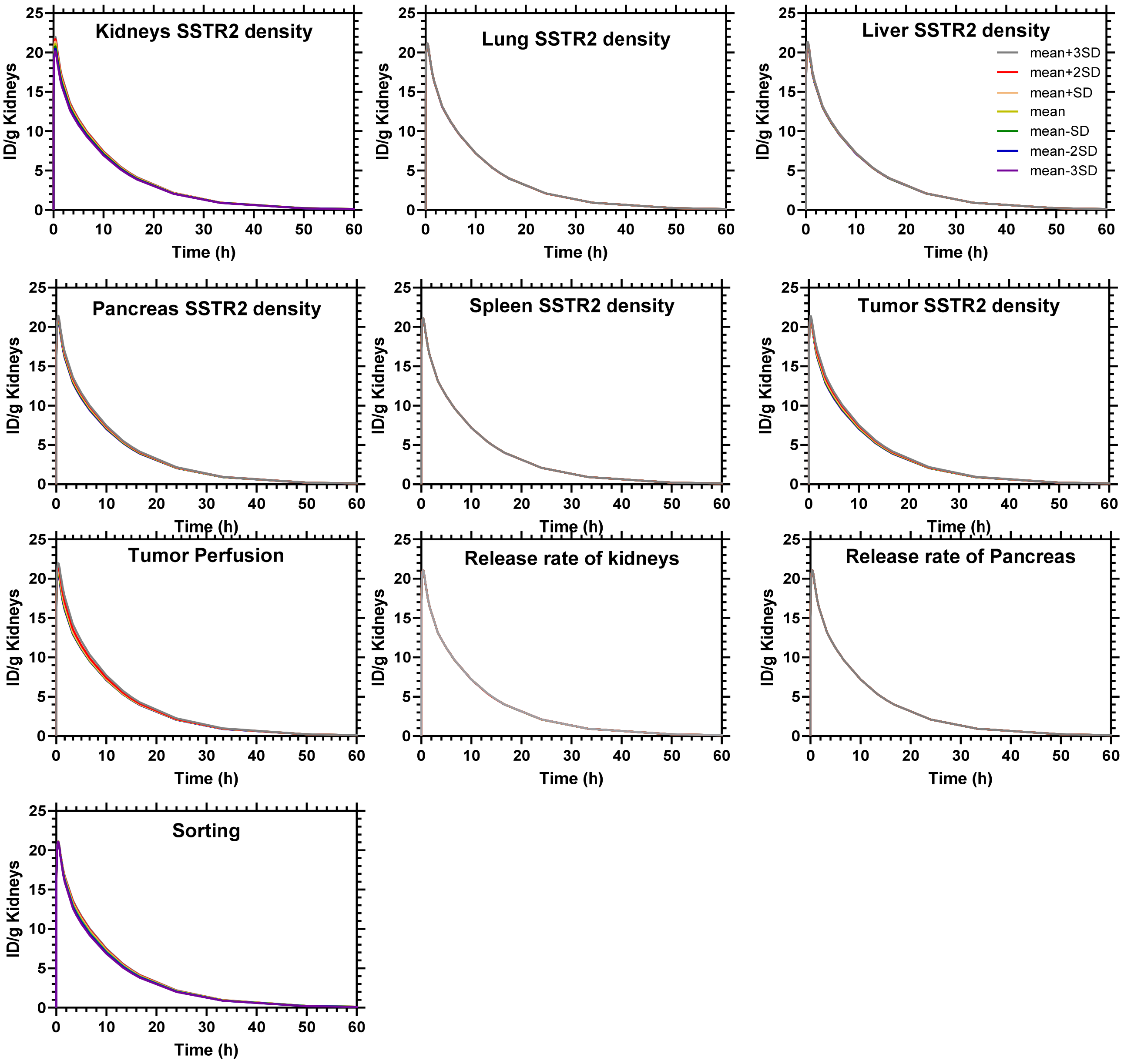

Figure S2. Kidneys sensitivity plots. The time course of the injected dose per gram (ID/g) of kidneys was investigated using a plausible range for the fitted input parameters (mean ± 0, 1, 2 and 3 SD). The uncertainty of the fitted pharmacokinetic parameters do not have a large influence on the ID/g time course of the kidneys.

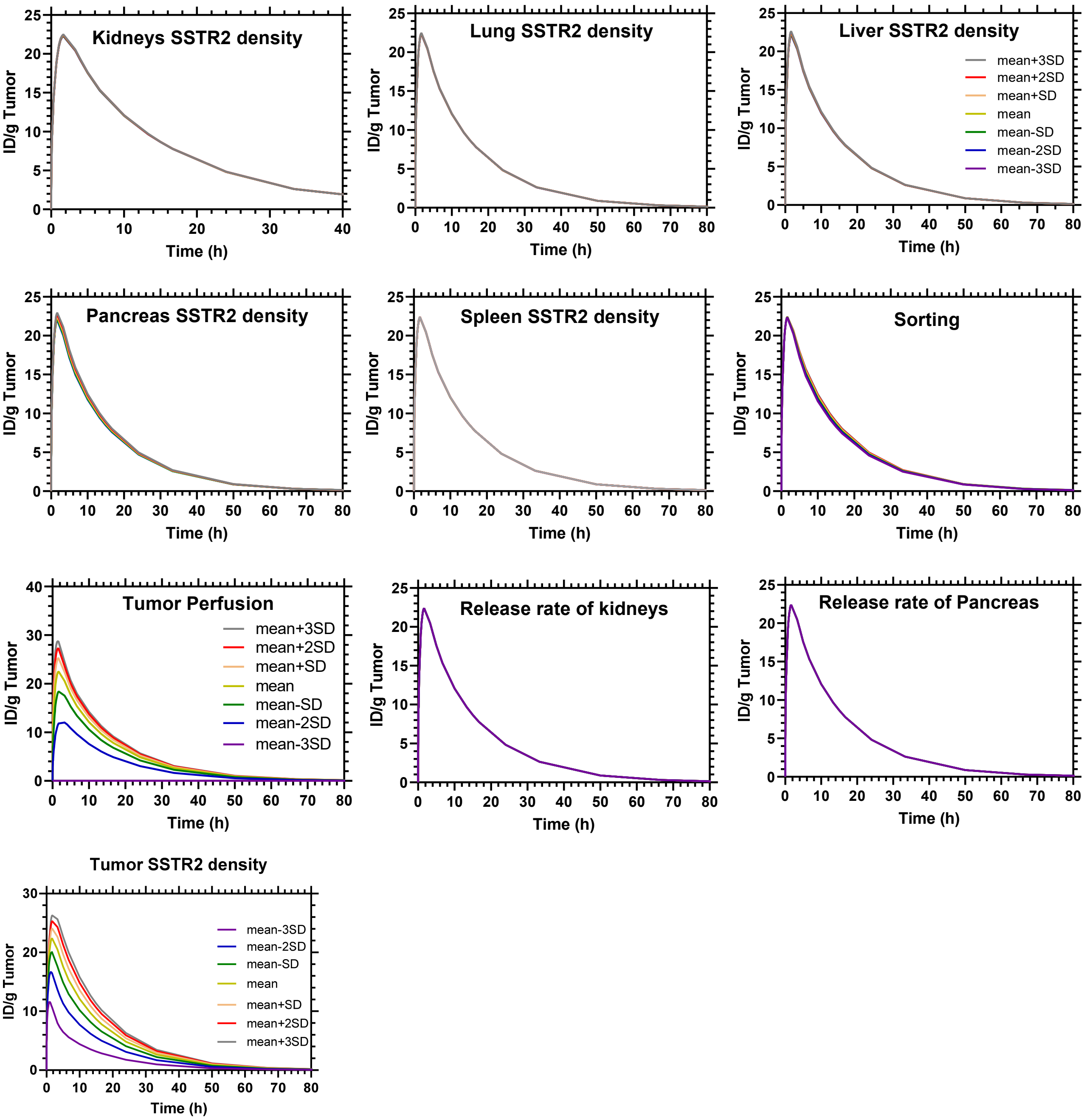

Figure S3. Tumor sensitivity plots. The time course of the injected dose per gram (ID/g) of tumor was investigated using a plausible range for the fitted input parameters (mean ± 0, 1, 2 and 3 SD). The SSTR2 density and the perfusion have a large influence on the time course of ID/g, which is related to their high uncertainty.

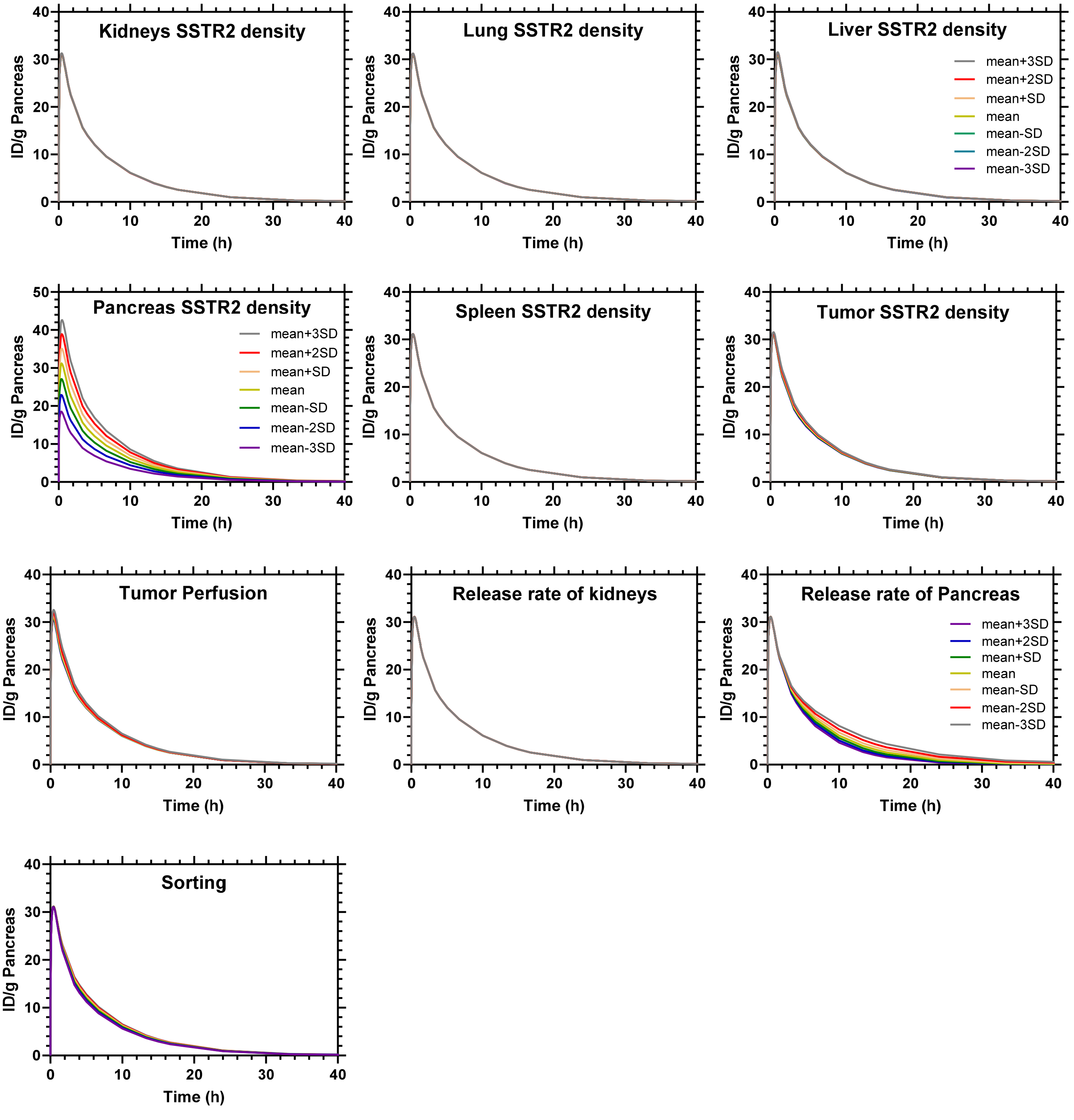

Figure S4. Pancreas sensitivity plots. The time course of the injected dose per gram (ID/g) of pancreas was investigated using a plausible range for the fitted input parameters (mean ± 0, 1, 2 and 3 SD). SSTR2 density and release rate of pancreas have a large influence on ID/g time course of the pancreas.

Supplement E: Evaluation of the Predictive Performance of the ^{212}Pb-SSTA-PBPK Model

Model parameters were fitted to the biokinetic data of the main biodistribution study performed by Stallons et al. (Figure 1 [7]). An initial evaluation for the predictive capacity of the developed model can be performed by simulating other studies shown in Supplemental Figure S2.A [7] (Effect of sex on ^{212}Pb-DOTAMTATE), Supplemental Figure S2.B [7] (Effect of specific activity on ^{212}Pb-DOTAMTATE) and Supplemental Figure S7 [7] (Biodistribution of ^{203}Pb-DOTAMTATE). For fitting or validation, important information required in addition to biokinetic data are the injected amount, activity and tumor volume. Unfortunately, the only biodistribution study in the above-mentioned publication that provides all these three prerequisites is in Figure 1 [7], whose data have been fitted. In addition, the animal model used in Figure 1 [7] (Athymic nude mice) differs from that used in Supplemental Figure S2 and Figure S7 [7] (CD-1 mice), a difference that certainly affects the measured biodistribution.

Nevertheless, based on assumptions described below, the predictive capacity of the ^{212}Pb-PBPK model was investigated in non-tumor-bearing mice. Thus, errors related to the non-specified tumor volume were avoided, as this has a considerable impact on biodistribution (sink effect) [20]. Accordingly, the biodistributions in Supplemental Figure S2.A and Figure S7 [7], that is, the effect of sex and the biodistribution of the imaging surrogate ^{203}Pb-DOTAMTATE, respectively, were simulated as follows:

A. The effect of sex on the biodistribution of ^{212}Pb-DOTAMTATE:

Since the injected amount and activity are not explicitly given, the injected amount was assumed the same as the amount used previously in fitting the main biodistribution of ^{212}Pb-DOTAMTATE in Figure 1 [7] (5 μCi with specific activity of 2.4 μCi/ng). The ^{212}Pb-PBPK model was adapted to simulate a non-tumor-bearing mouse by setting the perfusion rate to the tumor to zero. The simulation results are shown in Table S3.

Table S3. The predicted values of %ID/g for relevant tissues after injecting 5 μCi of ^{212}Pb-DOTAMTATE with a specific activity of 2.4 μCi/ng.

Time (h)	Kidneys	Liver	Lung	Pancreas	Spleen	Tumor	GI	Heart	Muscle	Brain
4	17.3	0.8	6.8	19.8	2.9	0.0	9.2	0.1	0.1	0.0
24	10.7	0.6	6.2	4.9	2.6	0.0	2.3	0.0	0.0	0.0

The simulated biodistribution of ^{212}Pb-DOTAMTATE agrees better with the measured biodistribution in the kidneys of males than with that of females at the investigated time points [7]. This can be attributed to the use of volumes of organs and sub-organs of a standard mouse as described in Supplemental table S1. Taking into account that the standard deviations for the published biodistributions in male and female mice are high, the simulation results of the model agree with the measurements in kidneys.

Using the same argument, the model simulations for pancreas and lung are also in good agreement with the published results in Supplemental Figure S2 [7]. The uptakes in liver and spleen will be discussed below.

B. The biodistribution of the theranostic pair $^{212/203}$Pb-DOTAMTATE:

The virtual non-tumor-bearing mouse was adapted to simulate the imaging surrogate ^{203}Pb-DOTAMTATE by changing the physical decay rate. The injected amount of ^{203}Pb-DOTAMTATE was also assumed to be equal to the amount used in Figure 1. As expected, the decay-corrected results are the same as in Table 1 and that agrees with the

conclusion of Stallons et al. Notably, Supplemental Figure S7 shows a systematic difference in the uptakes of tissues: ^{203}Pb-DOTAMTATE is slightly higher than ^{212}Pb-DOTAMTATE in all tissues except kidneys, where it is vice versa. As the injected activities and amounts of $^{212/203}$Pb-DOTAMTATE are not reported, a full interpretation of the measured %ID/g is not possible. The published biodistributions of ^{212}Pb-DOTAMTATE in non-tumor-bearing mice are also inconsistent (Supplemental Figure S2.A and Figure S7, particularly for liver, spleen, and lung. Although the predicted uptakes in liver and spleen are consistent with the published results in Supplemental Figure S7, they differ from those in Supplemental Figure S2A. Nevertheless, the model adequately describes the uptakes of $^{212/203}$Pb-DOTAMTATE in kidneys, liver and spleen in Supplemental Figure S7.
